# Cellular dynamics of the SecA ATPase at the single molecule level

**DOI:** 10.1038/s41598-021-81081-2

**Published:** 2021-01-14

**Authors:** Anne-Bart Seinen, Dian Spakman, Antoine M. van Oijen, Arnold J. M. Driessen

**Affiliations:** 1 Department of Molecular Microbiology, Groningen Biomolecular Sciences and Biotechnology Institute, and the Zernike Institute for Advanced Materials, University of Groningen, Groningen, The Netherlands; 2 School of Chemistry, University of Wollongong, Wollongong, Australia; 3 Present Address: AMOLF, Science Park 104, 1098 XG Amsterdam, The Netherlands

**Keywords:** Cellular imaging, Protein transport

## Abstract

In bacteria, the SecA ATPase provides the driving force for protein secretion via the SecYEG translocon. While the dynamic interplay between SecA and SecYEG in translocation is widely appreciated, it is not clear how SecA associates with the translocon in the crowded cellular environment. We use super-resolution microscopy to directly visualize the dynamics of SecA in *Escherichia coli* at the single-molecule level. We find that SecA is predominantly associated with and evenly distributed along the cytoplasmic membrane as a homodimer, with only a minor cytosolic fraction. SecA moves along the cell membrane as three distinct but interconvertible diffusional populations: (1) A state loosely associated with the membrane, (2) an integral membrane form, and (3) a temporarily immobile form. Disruption of the proton-motive-force, which is essential for protein secretion, re-localizes a significant portion of SecA to the cytoplasm and results in the transient location of SecA at specific locations at the membrane. The data support a model in which SecA diffuses along the membrane surface to gain access to the SecYEG translocon.

## Introduction

Translocation of proteins across membranes is an essential process in all living cells. In bacteria, secretory proteins (preproteins) are synthesized at ribosomes in the cytosol, targeted to the cytoplasmic membrane by molecular chaperones such as SecB, and translocated across this membrane by a conserved secretion (Sec) complex. Its central component is the heterotrimeric SecYEG complex, which forms a protein conducting channel in the cytoplasmic membrane^1^. Ancillary components like the heterotrimeric SecDFyajC complex or the ATPase SecA^2,3^ associate with the SecYEG translocon to facilitate protein translocation. SecA is a motor protein that utilizes ATP to mediate the translocation of unfolded preproteins through the SecYEG channel into the periplasm^4^, a process that is stimulated by SecDFyajC and the proton motive force (PMF)^5–7^.

SecA comprises several highly conserved structural and functional domains involved in nucleotide and preprotein binding^8–11^. Despite these structural insights, the exact molecular mechanism by which SecA mediates translocation is still poorly understood. Both monomeric and dimeric crystal structures of SecA have been described that differ in conformation and/or dimer interface^8,9,12–15^. Moreover, SecA is purified from cells in a dimeric form and in vitro translocation studies suggested that SecA is functional as a dimer^16–18^. Although SecA has been suggested to be functional as a monomer^19^, the mutation-induced monomerization of SecA is associated with a severe loss of activity^20,21^. Remarkably, in vivo, activity of the monomer can be restored when highly overexpressed and this is accompanied with a restoration of the dimeric state^18^. Since SecA is readily isolated from cellular lysates, a predominant cytosolic localization has been suggested^20,22–24^. However, some SecA is tightly bound to the membrane and is only released upon urea or carbonate extraction. The latter likely reflects a population of SecA that is bound to anionic phospholipids via its amphipathic N-terminus that penetrates the membrane^25^. The lipid interaction primes SecA for high-affinity binding to SecYEG^26^, lending strong support that the lipid-bound SecA is an intermediate in the functional cycle^27^. Furthermore, SecA also binds to ribosomes^28^ suggesting that there are at least four distinct populations of SecA in the cell.

Estimates on the exact number of SecA molecules per cell reported in literature vastly differ. Two studies cite on the basis of undocumented quantitative immunoblot analysis that SecA is present in *E. coli* cells at micromolar concentrations (5–8 µM)^19,29^. This would correspond to 8000–13,000 SecA copies per cell. Proteomic approaches report cellular copy numbers ranging from 46 to 987^30^, 518^31^, 500–1000^32^ and 2500–5000^33^. A single-cell FACS analysis suggest that SecA is a low abundance protein, with only 71 SecA molecules per cell^34^. In turn, ribosome profiling suggests a range of 1480–3987 molecules per cell per generation^35^ assuming a constant rate of translation. None of these methods, however, directly visualize the number of SecA copies per cell nor did they determine the exact cellular localization and distribution, the dynamics of localization and the quaternary state.

Several studies examined the cellular localization of SecA using conventional fluorescence microscopy in bacteria, indicating a membrane and cytosolic localization^23,24^. However, in recent years, developments in fluorescence microscopy paved the way for single-molecule visualization inside living cells. The high spatial and temporal resolution of this technique makes it possible to visualize biological processes with great detail and provides dynamic information on protein diffusion and localization in its native environment providing insights that cannot be gained by conventional fluorescence microscopy. In the present study, we used super-resolution fluorescence microscopy to examine the cellular concentration of SecA, its localization, its oligomeric state, and the dynamics of localization. By expressing functional SecA from its native locus as a fusion to a fluorescent protein marker and visualizing its fluorescence by using high-sensitivity fluorescence imaging, we demonstrate that in viable cells, SecA is a low abundance dimeric protein that is predominantly membrane-associated where it distributes over three distinct diffusional populations. The data support a model in which SecA diffuses along the membrane surface to gain access to the SecYEG translocon.

## Results

### Super-resolution localization and cellular distribution of the SecA ATPase

To study the cellular distribution of SecA in living *E. coli* cells, we performed PALM-type super-resolution imaging at the single-molecule level. To this end, the fluorescent proteins (FPs) Ypet and the photo-convertible mEos3.2 were functionally integrated into the genome at the *secA* locus (Supplementary Figures S1, S2 and S3) yielding C-terminal SecA fusions. We employed this region of SecA as it is flexible and directs away from the core structure^12,36,37^, while it can be deleted without activity loss^38^. Furthermore, it was previously used for plasmid-based SecA-GFP fusion expression^24^. Cells containing the chromosomal SecA-FP fusion constructs were viable with growth kinetics similar to the wild-type strain (Supplementary Figure S2A) which demonstrates the functionality of the SecA-FP fusion constructs. Quantification of the expression levels in a known concentration of cellular lysate, showed comparable to native SecA expression levels, for SecA-Ypet (102%) and SecA-mEos3.2 (91%) (Supplementary Figure S2B and S3). Furthermore, no degradation of SecA-Ypet or SecA-mEos3.2 occurred (Supplementary Figure S2B,C).

**Figure 1:**
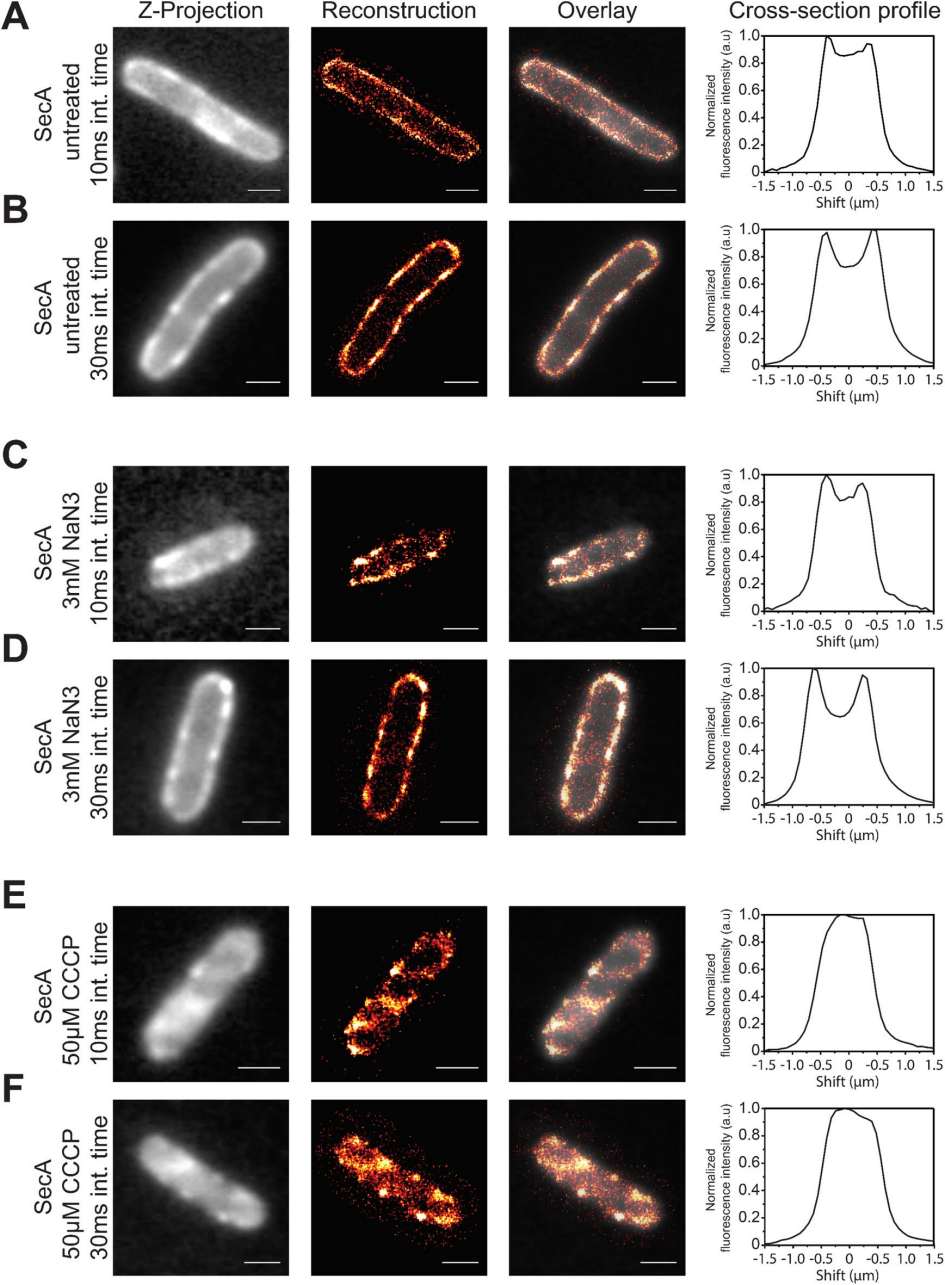
Super-resolution reconstructions and cellular distributions of SecA-Ypet under different conditions. (**A**,**B**) SecA under native conditions, (**C**,**D**) SecA-mediated translocation is blocked by 3 mM NaN_3_ (**E**,**F**) protein translocation is impaired due to collapse of the PMF by 50 µM CCCP. (**A**–**F**). Visible in the left panels (Z-projection) are the results of averaging the fluorescence in the first 7.5 s into one image with a single frame acquisition time of 10 ms (**A**,**C**,**E**) and 30.5 ms (**B**,**D**,**F**). Signals observed in the cytosol are a combination of fast moving cytosolic (auto)fluorescence and out of focus signals originating from molecules within the cytoplasmic membrane on the axial axis. Second panel (Reconstruction), super-resolution reconstruction images showing signal detected at the cytoplasmic membrane. Colors indicate the frequency and accuracy of signal observed at the coordinate. Red indicating a low fit accuracy and/or frequency, whereas white signifies a high fit accuracy and/or frequency of fluorescence observed at that location. Third panel (Overlay), a merge of the super-resolution reconstruction with Z-projection to clarify the localization of SecA-Ypet. Fourth panel (Cross-section profile), a short axis cross section profile of the normalized fluorescence intensity distribution of each cell. Scale bar is 1 µm.

**Figure 2:**
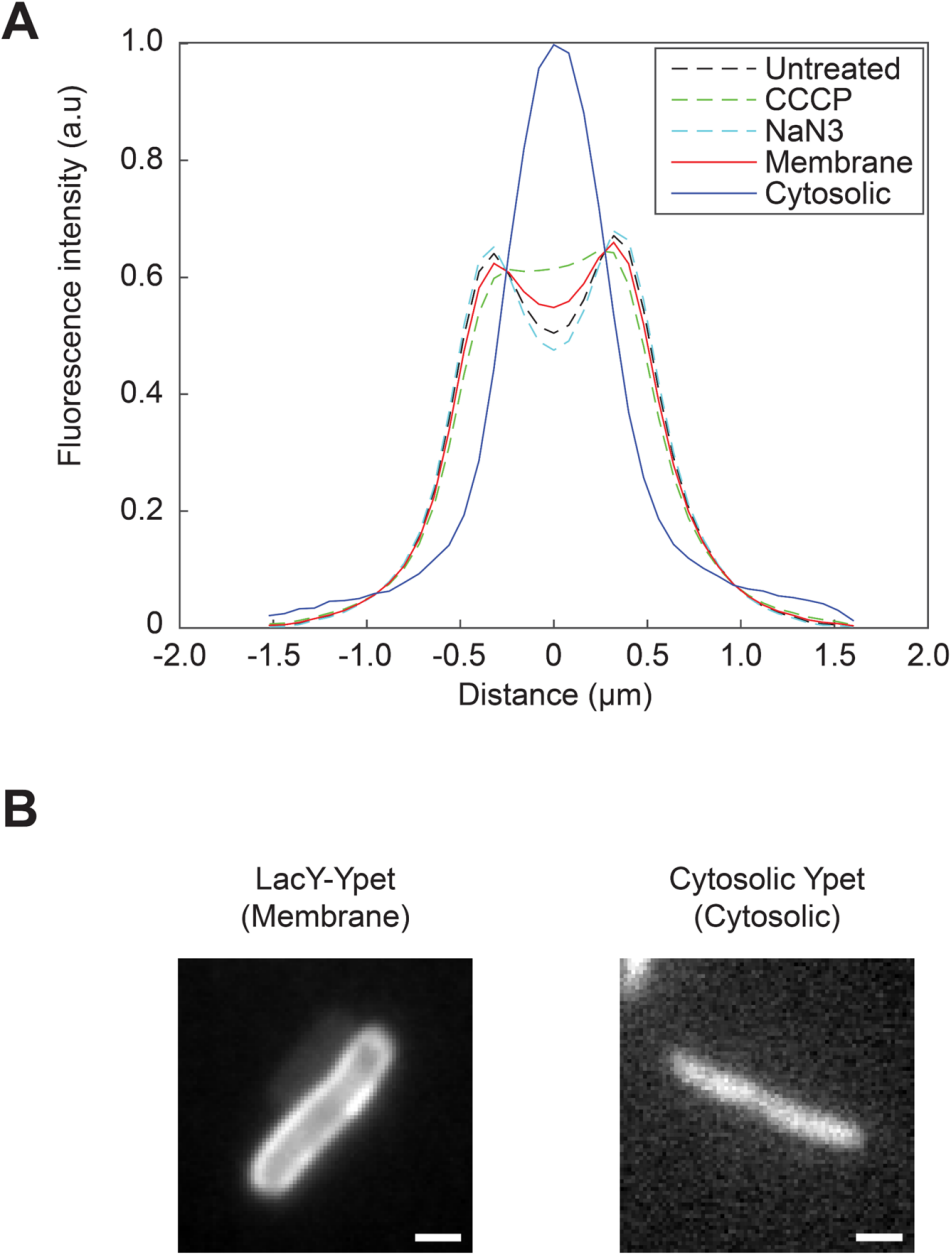
Cellular distribution profiles. (**A**) Cellular distribution profiles of the fluorescence for the membrane protein LacY-Ypet (Membrane, red solid line), cytosolic protein Ypet (Cytosolic, blue solid line) and SecA under native conditions (Black dashed line), treatment with 3 mM NaN_3_ (Green dashed line) and treatment with 50 µM CCCP (Cyan dashed lines). (**B**) Z-projections of the membrane marker LacY-Ypet and cytosolic marker cytosolic Ypet. Plots are the result of averaging the fluorescence in the first 10 s into one image with a single frame acquisition time of 30 ms. Scale bar is 1 µm.

Using super-resolution microscopy, the spatial distribution of SecA-Ypet was visualized in single cells (Supplementary Figure S4) with a localization accuracy of 10–20 nm, well below the diffraction limit. Reconstructing the fluorescent signals of cells grown under native expression conditions showed an enrichment of fluorescence at the cytoplasmic membrane (Fig. 1A,B). The recorded fluorescence and the localized molecules are distributed along the cytoplasmic membrane (Fig. 1A,B; reconstruction, cross-section profile), indicating that SecA molecules are moving along the membrane at a time scale faster than the time needed to obtain all the localization data, but slower than the timescale needed for a single image (10 or 30 ms). Small regions with an increased detection frequency are observed, and could indicate locations of SecA mediated protein translocation. Hardly any cytosolic fluorescence was detected using this technique. Whilst reconstructing the fluorescence is an excellent technique for localization, it cannot provide information about proteins that are rapidly diffusing in the cytosol. Therefore, cellular fluorescence distribution profiles of an established membrane protein, i.e., LacY-Ypet (Fig. 2B), and a cytosolic reference, Ypet (Fig. 2B), were used to deconvolve the SecA fluorescence intensity profile (Fig. 2A and Supplementary Figure S5). Deconvolution indicated that the majority of SecA (> 90%) is associated with the cytoplasmic membrane, implying that the cytosolic pool of SecA is much smaller than previously suggested by cell lysis and subcellular fractionation.

To determine if the inhibition of protein translocation affects the SecA localization, *E. coli* cells were treated with sub-lethal concentrations of the PMF uncoupler carbonyl cyanide 3-chlorophenylhydrazone (CCCP) and the SecA ATPase inhibitor sodium azide (NaN_3_). Reconstructions show little difference in SecA localization when cells are treated with sodium azide (Fig. 1C,D) which is also evident by a similar deconvolution of the cross-section profile (Fig. 2A). In contrast, incubation with sublethal CCCP concentrations resulted in highly localized foci (Fig. 1E,F). This behavior could indicate stalling of translocation due to the lack of a PMF and rescue attempts from SecA to recover from this state. Moreover, CCCP also induced a re-localization of SecA within the cell, now also showing significant cytosolic localization (Fig. 2A). Deconvolution confirms a more distinct cytosolic population (~ 14%). Treatment of cells expressing LacY-Ypet did not show a relocalization nor a difference in localization compared to normal LacY-Ypet expression (Supplementary Figure S6).

### Intracellular concentration of SecA obtained from single cells

**Figure 3:**
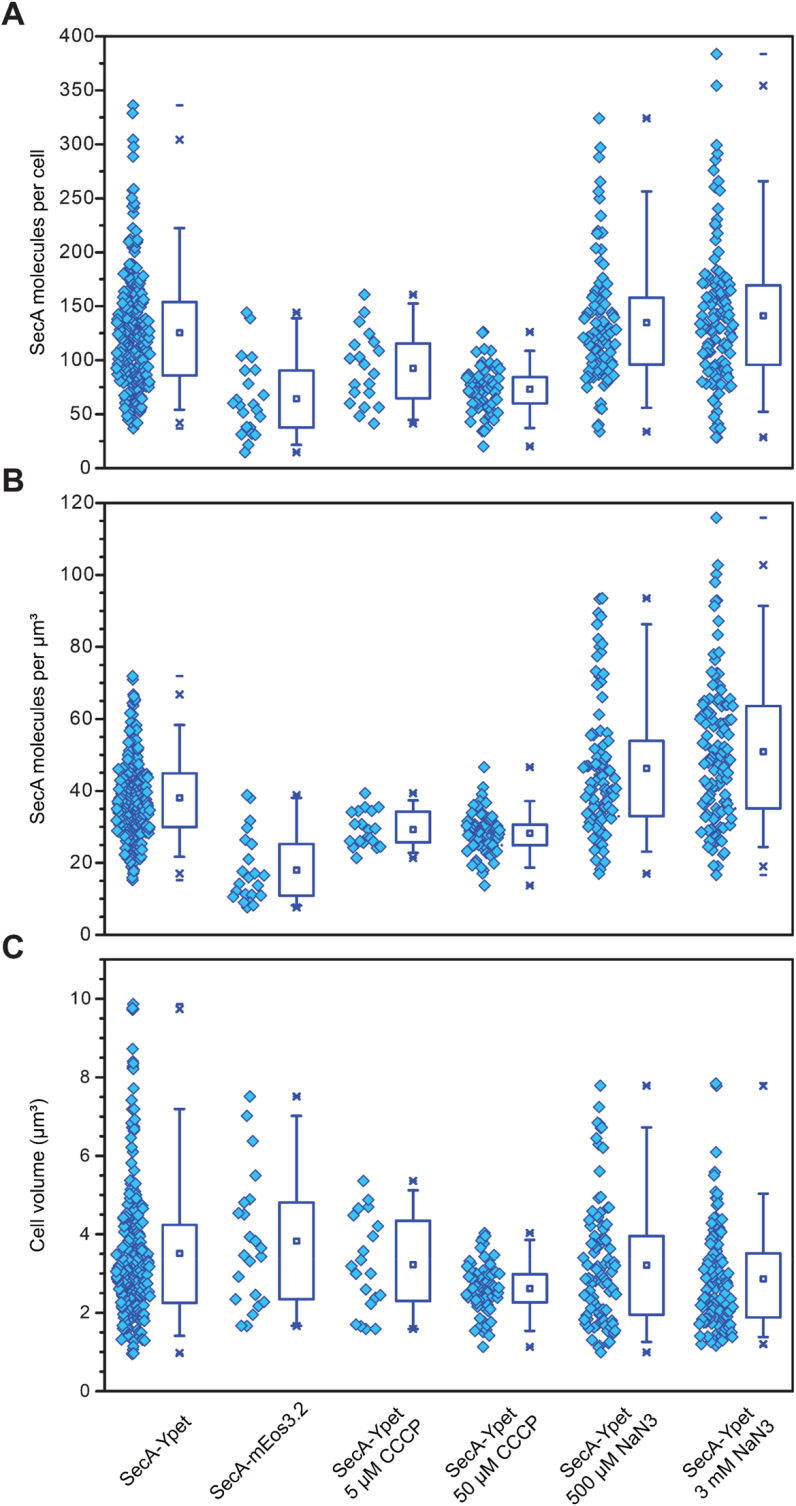
Cellular SecA copy numbers under different conditions. Plotted per condition are the values for each individual cell (left, open diamond) and corresponding boxplot (right) showing the lower 25% and upper 75% quartile with mean indicated by (open square). (**A**) SecA molecules calculated per cell. (**B**) SecA molecules per cubic micrometer or femtoliter, and estimated cell volumes (**C**). Whiskers indicate the lower 5% and upper 95% fence.

Next we determined the concentration of SecA within individual cells. To this end, the single-molecule intensity of Ypet was determined from fluorescent molecules spatially well separated in the last seconds prior to complete photobleaching. Due to the stochastic nature of bleaching, these molecules are single-molecules and by plotting the calculated intensities in distributions, the largest population represent the intensity of a single fluorescence molecule (Supplementary Figure S7). Next, the cellular copy number of SecA was calculated under both native and stress conditions by integrating the total cellular fluorescence intensity and dividing this number by the single-molecule intensity and the cellular volume. The boxplots in Fig. 3 show the combined SecA copy numbers, molecules per µm^3^ and cell volume under different conditions obtained from multiple independent microscopy experiments. When grown under native conditions, the majority of the exponentially growing *E. coli* cells expressed between 86 and 154 SecA-Ypet molecules (Fig. 3, SecA-Ypet boxplot interquartile range (IQR) and Table 1). The remaining cells expressed SecA-Ypet in a range of 37–336 molecules per cell. To minimize the variation due to different cell volumes, the copy number for each cell was divided by the volume of that particular cell (Table 1). This procedure resulted in an average of 38 SecA molecules per µm^3^ centered around a range of 30–45 SecA molecules per µm^3^, with a minimum and maximum number of 15–72 SecA molecules per µm^3^ (Fig. 3, SecA-Ypet boxplot IQR and Table 1). To verify the obtained observations with a different fluorophore, the green-to-red photo convertible mEos3.2 was used (Fig. 3, SecA-mEos3.2). The majority of exponentially growing cells, express between 38 and 90 SecA-mEos3.2 molecules (Fig. 3, SecA-mEos3.2 boxplot IQR and Table 1). The remainder of cells expressed SecA-mEos3.2 in a range of 15–144 molecules per cell. The decrease compared to the SecA-Ypet strain results from the photo conversion efficiency, as not all green fluorescent molecules are switched to red upon irradiation. A recent study on photo activation efficiencies showed an average conversion for mEos3.2 of 42 ± 9%^39^ which is consistent with the discrepancy in the copy number between Ypet and mEos3.2. Taken from these results, the copy number of SecA under native conditions ranges between ~ 37 and ~ 336 molecules per cell, corresponding to a cellular concentration of approximately 23–207 nM (assuming average cell volume 3 µm^3^).

**Table 1:** SecA cellular copy number ranges under native and stressed conditions.

Strain	Q1	Q3	Lowest detected copies per cell	Highest detected copies per cell	Cell volume (µm^3^)	Average copy number per cell	Average molecules per µm^3^	*n*
SecA-Ypet	86	154	37	336	3.51	126	38	255
+ 5 µM CCCP	65	116	41	161	3.23	93	29	20
+ 50 µM CCCP	60	84	20	126	2.62	73	28	72
+ 500 µM NaN_3_	96	158	34	324	3.21	135	46	95
+ 3 mM NaN_3_	96	169	28	384	2.86	141	51	122
SecA-mEos3.2	38	90	15	144	3.53	64	16	102

To investigate the effect on the intracellular concentration of SecA by blocking protein translocation, cells were incubated for 30 min in the presence of 5 µM CCCP. Under those conditions there was a slight decrease in SecA numbers compared to the untreated strain, to a range of 65–116 SecA-Ypet molecules per cell (Fig. 3, 5 µM CCCP boxplot IQR and Table 1). Whilst the lowest detected copy number of 41 is comparable to that of the untreated condition, the highest detected number decreased twofold to 161 molecules per cell. Increasing the CCCP concentration to 50 µM significantly affected the average cell volume, cells shrank from 3.5 ± 0.1 SEM to 2.6 ± 0.1 SEM µm^3^, while the SecA numbers decreased further to a range of 60–84 molecules (Fig. 3, 50 µM CCCP boxplot IQR and Table 1). Moreover, the total spread of molecules per cell decreased significantly compared to the untreated condition to a minimum of 20 and a maximum of 126 molecules per cell. This decrease in SecA numbers is most likely due to the effect of CCCP on the metabolic state of the cell. After 60 min of incubation, most CCCP treated cells had lysed.

Sodium azide (NaN_3_) is a well-known inhibitor of SecA and blocks translocation both in vivo and in vitro^7,40^. In vivo, NaN_3_ causes an upregulation of the *secA* gene via a transcriptional feedback by SecM^7^. Indeed, an increase in SecA concentration to a range of 96–158 molecules per cell was observed when cells were incubated in the presence of 500 µM NaN_3_ (Fig. 3, SecA-Ypet 500 µM NaN_3_ IQR and Table 1). The upregulation was slightly higher when cells were grown in the presence of 3 mM NaN_3_, increasing to an average Third Quartile (Q3) of 169 (Fig. 3, SecA-Ypet 3 mM NaN_3_ IQR and Table 1). Though the upregulation of SecA observed here is weaker than reported before using transcriptional reporters and bulk recordings^7^, shorter preincubation with NaN_3_ were used for the imaging to prevent cells from dying too quickly during measurements.

### Oligomeric state of SecA in cells

**Figure 4:**
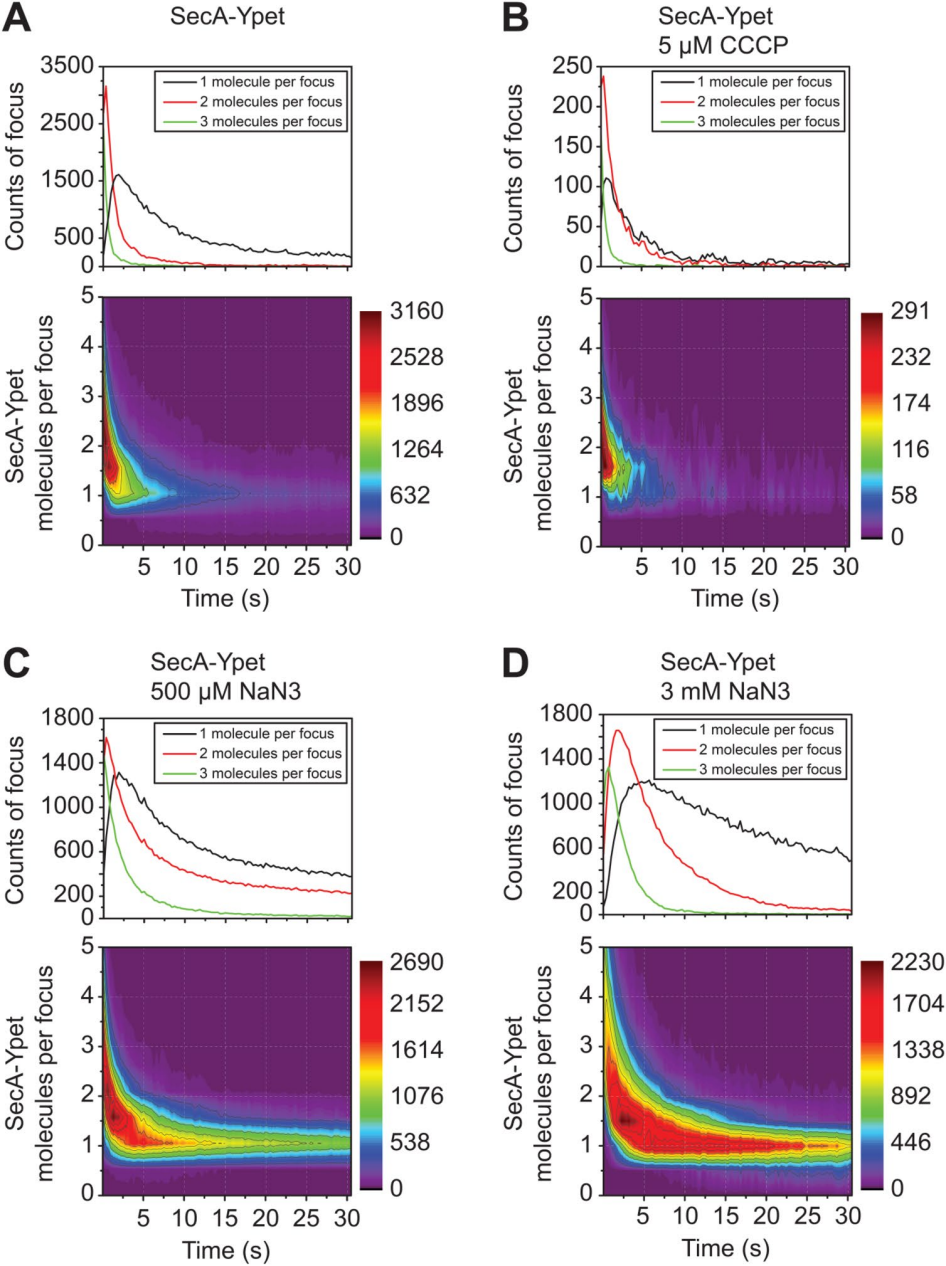
Oligomeric state of SecA under different conditions subjected to bleaching as a function of time. The oligomeric state of SecA-Ypet is presented as a heat map ranging from dark purple (lowest occurrence) to dark red (highest occurrence) and line plot for the total count foci with single, double or triple molecules. Initially, under native conditions (**A**), a population around two SecA-FP molecules per focus is observed, indicating the dimeric state of SecA. (**B**) Treatment with 5 µM CCCP did not change the dimeric state, as seen by the hot spot around the 2 molecules per focus. (**C**,**D**) Treatment of 500 µM (**C**) and 3 mM NaN_3_ increased the SecA expression, as observed by increased detections at later time points. Initially, the number of molecules per focus is increased to higher oligomers as a result of false-readouts due to this increased copy number. Black contour lines indicate major levels of frequencies as indicated by the numbers in the legend.

To investigate the oligomeric state of individual SecA molecules in living cells, foci intensities in the unprocessed image were analyzed. Based on a single-molecule intensity, a minimal fixed gray value threshold was used to select foci. Next, the number of molecules per focus were determined based on the emitted fluorescence intensity divided by the intensity value of a single Ypet molecule. We found that the majority of foci in the SecA-Ypet expressing strain initially consist of two molecules as indicated in the heat map by the dark red population (Fig. 4A). The less frequent higher oligomeric states (line plot, green line) seen at this time point are caused by the initial high background fluorescence originating from overlapping PSF from foci out of the focal plane. Moreover, hardly any fluorescent monomeric species are observed at these early stages of bleaching. The total cell fluorescence and number of molecules per foci decreases in time due to bleaching, gradually transitioning to single-molecules as indicated by the light blue colors. Also, the foci intensities from the SecA-mEos3.2 expressing strain was measured in time (Supplementary Figure S8A). Due to the photo conversion efficiency, approximately 54% of the total mEos3.2 molecules were fluorescent, assuming the copy number obtained from the Ypet strain is the total quantity, the chance of observing dimeric foci with mEos3.2 are drastically decreased. Calculation of the number of possible combinations of fluorescent and bleached molecules, however, confirms the dimeric state of SecA-Ypet (Supplementary Figure S8).

Treatment with CCCP has little effect on the oligomeric state of SecA (Fig. 4B). In contrast, cells treated with 500 µM NaN_3_ show an increase in oligomers initially and over time (Fig. 4C). Increasing the NaN_3_ concentration to 3 mM caused a further increase of SecA oligomers (Fig. 4D). The spread of the oligomeric states is higher and also the bleaching occurs over an extended time. This is likely due to an elevated synthesis rate of SecA in the presence of NaN_3_, leading to an increased detection of newly synthesized proteins at the later time points. Taken together, these observations indicate dimerization as an intrinsic SecA protein property in cells, and show that NaN_3_ inhibition induces SecA aggregation into higher membrane-bound oligomers.

### Membrane diffusion rates of SecA

**Figure 5:**
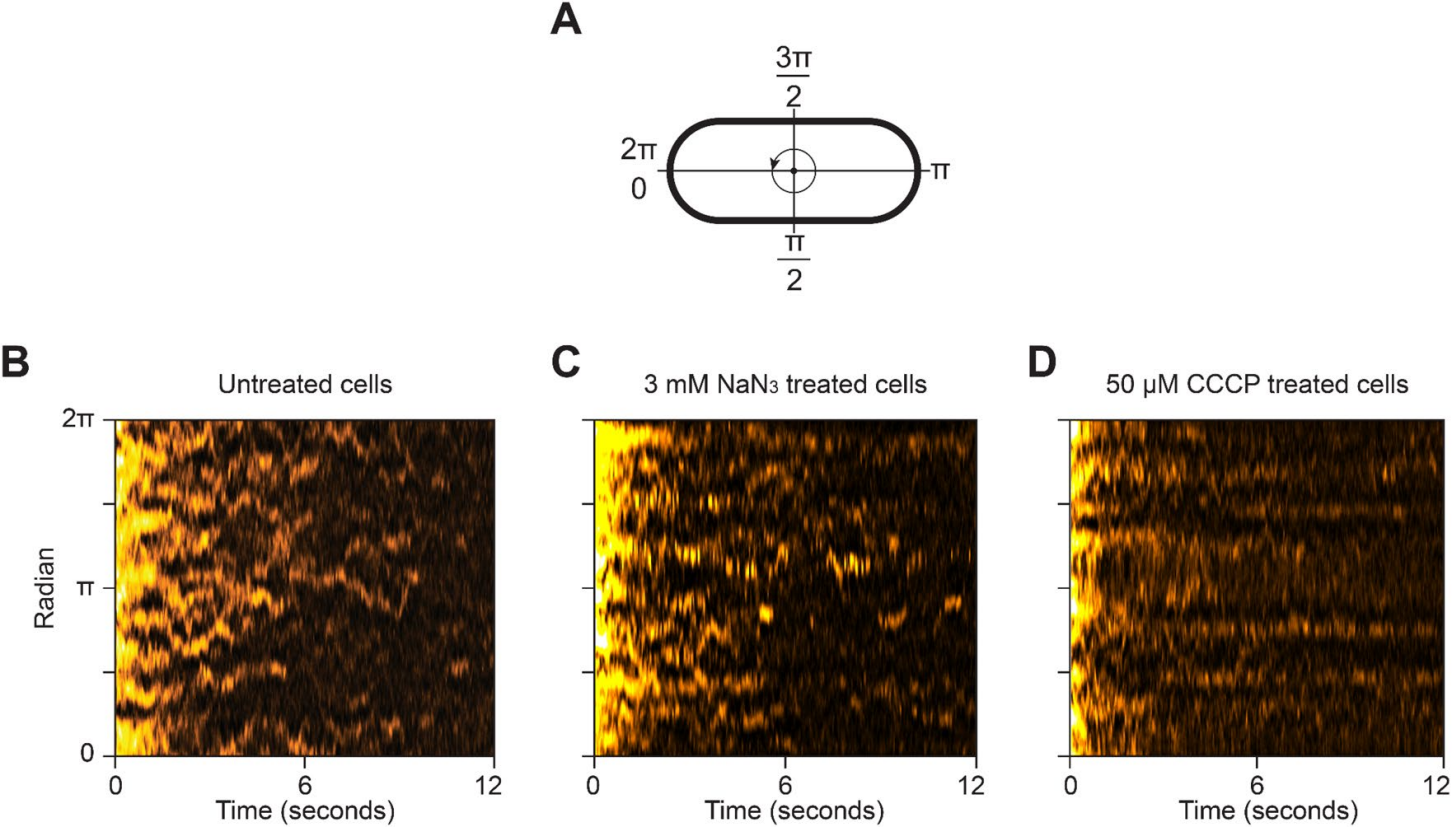
In vivo motion of SecA at the cytoplasmic membrane. (**A**) model of a cell outline with polar coordinates indicating the radians displayed at the kymographs. (**B**–**D**) Kymographs of the SecA-Ypet fluorescence along the cytoplasmic membrane under native and treated conditions. Movement of foci along the membrane results in diagonal lines where reoccurrences at the same location over time appears as horizontal lines.

The motion of the SecA-Ypet molecules along the cytoplasmic membrane is most easily observed in kymographs obtained from single cells (Fig. 5). For this purpose, the fluorescence of the SecA particles along the radial direction of the cytoplasmic membrane are plotted as a function of time (Fig. 5A). Moving SecA particles in untreated cells appear as erratic diagonal lines in the kymograph (Fig. 5B), corresponding to a highly dynamic motion along the cytoplasmic membrane. The kymograph for cells treated with 3 mM sodium azide did not change significantly from the untreated graph (Fig. 5C). However, treatment with 50 µM CCCP changed the erratic pattern to horizontal lines (Fig. 5D), indicating that SecA molecules stay or return to the same location in time, which corresponds to the highly localized spots observed with the super-resolution reconstructions (Fig. 1E,F). A more detailed analysis of these locations showed that these foci originate from very short reoccurrences of fluorescence (average 4 frames, ~ 122 ms on average per detection) at the same location over time (Supplementary Figure S9).

**Figure 6:**
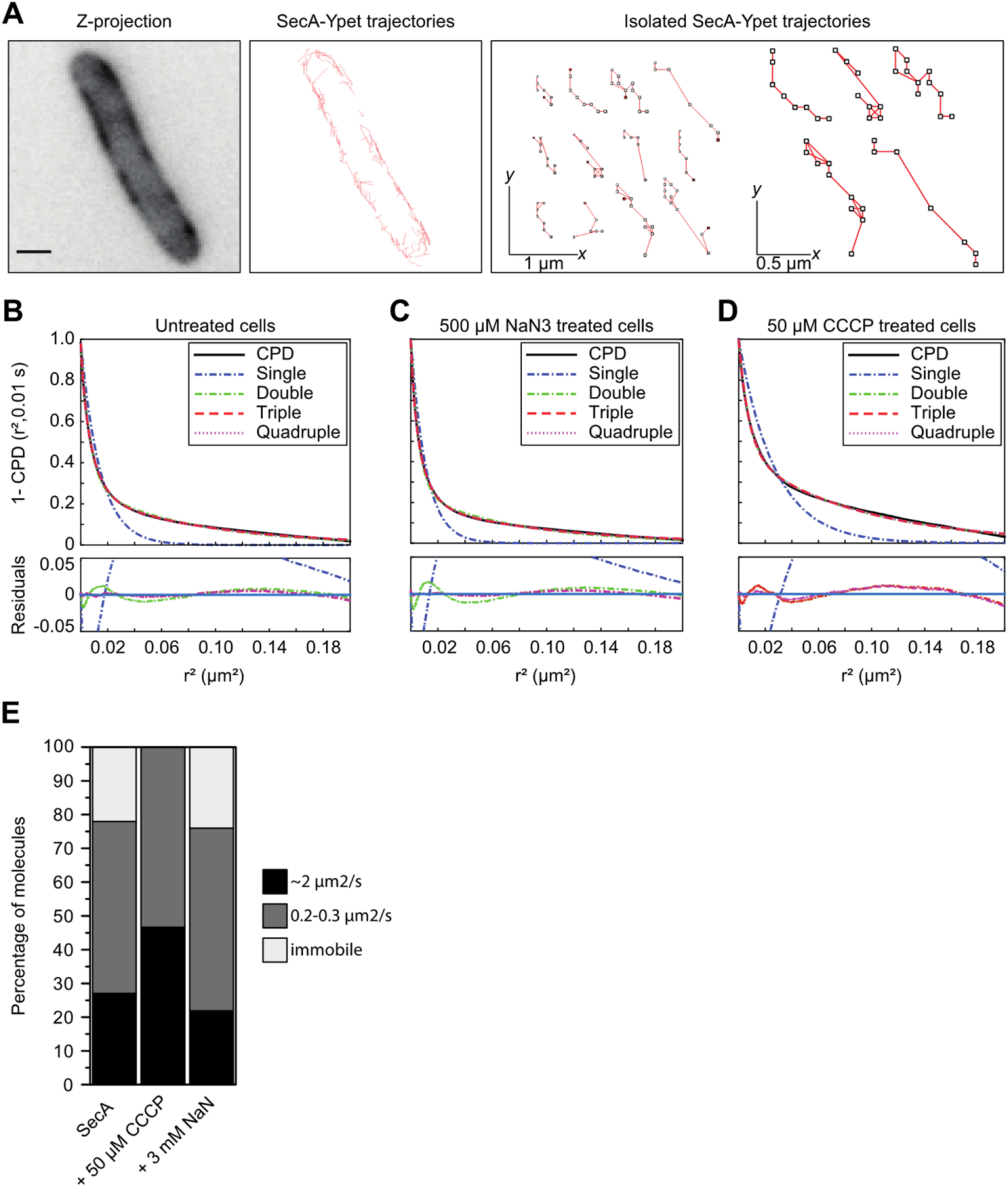
In vivo SecA-Ypet trajectories and diffusion curves. Scale bar is 1 µm. (**A**) Trajectories resulting from in vivo particle tracking display diffusion alongside the cytoplasmic membrane as well as static localization. (**B**–**D**, CDF, solid black line) reveal multiple populations (**B**–**D**, single, double and triple exponential CPD models, dashed lines) of different diffusion rates explaining the observed trajectories. CDF of untreated cells (**B**) and incubated with 3 mM NaN_3_ (**C**) are best described by a triple exponential CPD model. Indicating three diffusive populations. (**D**) CDF of cells incubated with 50 µM CCCP fits best to a double exponential CPD model, indicating two diffusive populations. (**E**) In vivo SecA-Ypet diffusion rates under native and protein secretion impaired conditions. Stacked bar chart summarizing the diffusion rates obtained from the CPD analysis. Under native conditions and impaired SecA-mediated protein secretion (+ 3 mM NaN_3_), three populations with comparable diffusion rates are observed. A significant change in the diffusive behavior of SecA is observed with the addition of 50 µM CCCP (+ 50 µM CCCP) as the immobile population is not detectable anymore. CPD indicates two populations remain.

To study the dynamics of SecA motion within cells in more detail, single-particle tracking of SecA-Yet molecules was used to calculate diffusion rates. The obtained trajectories showed that the behavior of the particles differs widely, ranging from highly confined movement to diffusion along the cytoplasmic membrane (Fig. 6A). Using mean square displacement (MSD) analysis, a diffusion coefficient for SecA-Ypet was obtained by collecting tracking data from 24 cells that were imaged with an acquisition time of 10 ms. From the SecA MSD data consisting of 450 trajectories we obtain a diffusion coefficient of 0.48 µm^2^·s^−1^ which corresponds to diffusion rates typical for integral membrane proteins (Table 2)^41–43^.

**Table 2:** Diffusion data of cytosolic and membrane(-bound) proteins and probes.

Protein/lipid dye	MM (kDa)	TM domains	Radius (nm)	Diffusion coefficient (µm^2^/s)	Fraction (%)	References
**Cytosolic**						
eYFP	26.7	–	3–4	7.08 ± 0.32		^66^
Crr-YFP	45.0	–	–	2.03 ± 0.05		^66^
HtpG-YFP	198.0	–	–	1.65 ± 0.07		^66^
SecA-Ypet	130.3	–	–	2.09 ± 0.10	27 ± 1.6	This study
+ 3 mM NaN_3_				2.19 ± 0.03	21 ± 1.1	This study
+ 50 µM CCCP				2.20 ± 0.04	48 ± 2.5	This study
**Membrane-bound**						
DiL-C12	–	–	–	0.365 ± 0.012		^45^
Bodipy FL-C12	–	–	–	1.502 ± 0.078		^45^
YedZ-eGFP	24.1	6	1.3	0.188 ± 0.004		^45^
CybB-eGFP	20.3	4	1.7	0.175 ± 0.008		^45^
GlpT-eGFP	50.3	12	2.0	0.153 ± 0.003		^45^
Tar(1-397)-YFP	142.3	4	–	0.217 ± 0.030		^66^
SecA-Ypet	130.3	–	–	0.209 ± 0.016	51 ± 3.3	This study
+ 3 mM NaN_3_				0.190 ± 0.010	55 ± 1.5	This study
+ 50 µM CCCP				0.160 ± 0.010	52 ± 2.5	This study

*MM* molecular mass.* TM domains *Transmembrane domains.

However, MSD analysis has its limitations as it assumes that each single-molecule behaves homogeneous. Therefore, the model cannot account for changes in behaviour throughout the trajectory, e.g. SecA binds or unbinds the SecYEG translocon. As an alternative approach to determining diffusion rates, we used the cumulative probability distribution (CPD) of step sizes^44–46^. As the CPD considers each step independently it can account for heterogeneity in diffusion but the diffusion rates are generalized, e.g. at any given time, a certain percentage of molecules diffuse with a certain rate. The cumulative probability distribution (CPD) is defined as the probability of a molecule staying within an area defined by a radius, r, after a given time, τ and provides diffusion data by fitting the cumulative probability distribution function (CPF) in Eq. (8) to the CPD curve.

1 $$P\left( {r^{2} ,\tau } \right) = 1 - \alpha e^{{\left( {\frac{{ - r^{2} }}{{r_{\alpha }^{2} + 4\sigma^{2} }}} \right)}} - \beta e^{{\left( {\frac{{ - r^{2} }}{{r_{\beta }^{2} + 4\sigma^{2} }}} \right)}} - \gamma e^{{\left( {\frac{{ - r^{2} }}{{r_{\gamma }^{2} + 4\sigma^{2} }}} \right)}}$$

Tracking SecA molecules in untreated cells, resulted in a CPD curve that was described best with a triple exponential CPF model (Eq. 1, Fig. 6B), as the residual sum of squares (RSS) did not decrease significantly after adding another component (RSS 0.24 $\pm { }0.03$ a.u.). Fitting the CPD curve to the multi-component CPF in Eq. (1), provided diffusion data for each population (α, β, γ) after a given time, τ. Where α, β, γ is the fraction of each population with the constraints that the sum of fractions cannot exceed 1, and the experimental localization accuracy (σ). The lateral diffusion coefficient for each population at each time point (τ) is given by ${\text{r}}_{{{\upalpha },{\upbeta },{\upgamma }}}^{2}$. The major population of SecA molecules in untreated cells (~ 51%) displayed a diffusion rate of 0.21 µm^2^ s^−1^, which is comparable to known diffusion rates from integral membrane proteins (Fig. 6E, SecA and Table 2). Such a diffusion coefficient would be consistent with SecA interacting with a membrane protein, possibly the SecYEG translocon which is its high affinity binding partner. The second population of SecA molecules (27%) showed a diffusion coefficient of 2.09 µm^2^ s^−1^, which is in line with diffusion rates of cytosolic proteins. Since the observed SecA signals originate close to the cytoplasmic membrane, this SecA population diffuses along the membrane interface. The remainder of the SecA molecules, 23%, were immobile—these SecA molecules might be bound to a large complex of the translocon and possibly ribosomes during active protein translocation to obtain temporally a negligible diffusion rate. To investigate the effect of an inhibited ATPase activity of SecA on the diffusion rates, cells were treated with 3 mM NaN_3_. Similar to untreated cells, step size data of these cells was best described by fitting a triple exponential CPF model (Fig. 6C, RSS 0.15 $\pm { }0.02$ a.u). The addition of NaN_3_ did not affect the diffusion rates significantly compared to the untreated cells. The largest population of 55% exhibit a diffusion coefficient of 0.19 µm^2^ s^−1^, while 21% had a diffusion rate of 2.19 µm^2^ s^−1^ and ~ 24% were immobile (Fig. 6E, + NaN_3_). However, dissipating the PMF with 50 µM CCCP had a major effect on the diffusion of SecA (Fig. 6D).The step size data did not fit to a three-component model (RSS 0.93 $\pm { }1.30{ }$ a.u.), but was described best by a double exponential CPF model (RSS 0.98 $\pm { }0.12$ a.u.). Compared to untreated cells, 52% of the SecA diffused with a rate of 0.16 µm^2^ s^−1^. Where ~ 48% of the molecules showed a diffusion rate of 2.20 µm^2^ s^−1^ (Fig. 6E, + CCCP). The lack of an apparent immobile population was striking as the reconstruction and kymographs data showed an increase in localization at specific spots. This absence is due to a lack of peak detections in consecutive frames, which indicates very short retention times of SecA at the sites of immobility discussed before.

## Discussion

The ATPase dependent motor protein SecA plays an essential role in protein translocation across the cytoplasmic membrane in bacteria. Despite the multitude of structural and biochemical data available, little is known about the dynamic behavior of SecA in living cells. Previous studies based on biochemical assays postulated that approximately half of the total cellular SecA proteins in *E. coli* are located in the cytosol^20,22–24^. Fluorescence microscopy reports suggest that SecA localizes in specific clusters at the cytoplasmic membrane in *Bacillus subtilis*, possibly in the shape of a spiral^24,47^. Using Super-resolution imaging in this study indicates that under native conditions, SecA is predominantly located at the cytoplasmic membrane where it is evenly distributed. Importantly, deconvolving fluorescent signals of a membrane and cytosolic protein, revealed that the cytosolic fraction of SecA is very small. The discrepancy with the fractionation is likely explained by release of weakly bound SecA from the lipid bilayer upon mechanical disruption of cells, resulting in an overestimation of the cytosolic SecA pool. Conventional fluorescence microscopy is often limited in spatial and temporal resolution and the projected nature of the images prevents an accurate estimate of the cytosolic pool. In the present study, in which short integration times lead to a high temporal resolution, we found no evidence for spiral formation of SecA in *E. coli*. An explanation for the spiral hypothesis suggested for *B. subtilis* could originate from the high mobility of SecA, where a low temporal resolution could lead to artifacts caused by fast diffusion SecA molecules^47^. A recent localization study of SecA in *Streptococcus agalactiae* localizes SecA predominantly at sites of ongoing peptidoglycan synthesis^48^. Super-resolution reconstruction of SecA does not show an equivalent situation at corresponding sites in exponentially growing *E. coli* cells. However, the dynamic data in our study are more in line with an immunogold electron microscopy study^49^ using antiserum against SecA showing also an even distribution of SecA in *Streptococcus pyogenes*. Nevertheless, as the latter study was carried out on fixed cells only a static picture of a highly mobile protein is obtained.

To gain more insight into the SecA localization pattern, we disrupted either the SecA ATPase activity via NaN_3_ or blocked protein translocation via the dissipation of the PMF. Addition of NaN_3_ did not change the localization pattern nor the cellular distribution as compared to the untreated cells. However, dissipating the PMF resulted in a markedly different localization pattern and a partially re-localization of SecA to the cytosol. The even distribution along the cytoplasmic membrane was replaced by a more localized pattern. Closer examining of these regions with an increased SecA detection, we found that these spots find their existence via reoccurrences of SecA molecules returning to the same location with very short retention times. We hypothesize that these spots are stalled translocons induced by uncoupling of the PMF and at which SecA attempts to rescue from this state by reinitiate a translocation step.

Widely varying numbers on the cellular concentration of SecA have been reported, ranging from 57 to 1794 as assessed from SecA-LacZ levels^32^, proteomics^30^ and FACS based single cell fluorescence^34^, up to a claimed high abundance of 8000–13,000 SecA copies per cell as determined using quantitative immunoblots^19,29^. Our in vivo single-cell approach yields a range of 37–336 molecules per cell with an average of 126 molecules per cell. These copy numbers correspond to a concentration of 23–207 nM per cell, which are in line with a single cell FACS^34^ and a recent proteomics study^30^. The numbers here are, however, an order lower compared to a ribosome profiling study^35^. It should be stressed that the latter methodology cannot measure protein copy numbers directly. Variations in translation efficiency, while translation rates of individual genes may not be constant during a complete generation, may affect the calculated protein count.

Our number might be influenced by a couple of factors; firstly, a possible transcription and translation effect due to the fusion construct, and secondly, the maturation of the fluorescent protein. These factors would ultimately lead to an underestimation of the actual number of SecA molecules per cell. To address these aspects, we used two different FPs for copy number determination, which both lead to a comparable number of SecA molecules per cell ruling out maturation as a major issue. A possible effect of the fusion construct on transcription and translation would have resulted in different protein levels, but western blots show similar levels of wild-type and fusion constructs. Therefore, we conclude that SecA concentrations in the cell are in the nanomolar range rather than in the micro molar range. Nanomolar concentrations of SecA are much easier to reconcile with the SecM-based regulatory mechanism of SecA expression than concentrations in the micromolar range. In vitro translocation assays suggest that the apparent *K*_*m*_ for SecA in translocation is about 100 nM^50^. Thus, an increased SecA expression as induced by a translocation stress via the SecM regulatory mechanism would only stimulate translocation when SecA levels are in the nanomolar range and would have little impact when SecA levels are already oversaturating at the upper micromolar concentration.

Using the in vivo single-molecule approach, we also addressed the oligomeric state of SecA in living cells. Under native conditions, we quantified the cytoplasmic membrane associated SecA-Ypet foci, which showed that particles residing at this membrane consisted of two molecules. Higher oligomeric states were detected, but in much lower numbers. The majority of these higher-order oligomerizations find their origin in overlapping single-molecule foci, leading to a higher intensity measured. Moreover, the mEos3.2 construct, confirmed a dimeric state of SecA. By employing a combinatorial analysis and an estimated 54% switching efficiency for SecA-mEos3.2 based on the deviation of numbers observed SecA-Ypet, only the dimeric state resulted in a maximum number of molecules detectable that lies in the range of the SecA-mEos3.2 copy number that was experimentally observed. Higher oligomeric states lead to an increase of the observable molecules, which would be too close to the detected copy number of SecA-Ypet. Therefore, we conclude that SecA is a homodimer associated with the cytoplasmic membrane. This contrasts an early in vitro report where a dissociation constant K_d_ of 0.1 µM was reported for SecA in solution based on gel filtration^51^, but is in line with the K_d_ of 0.74 nM estimated in a single-molecule study^50^.

**Figure 7:**
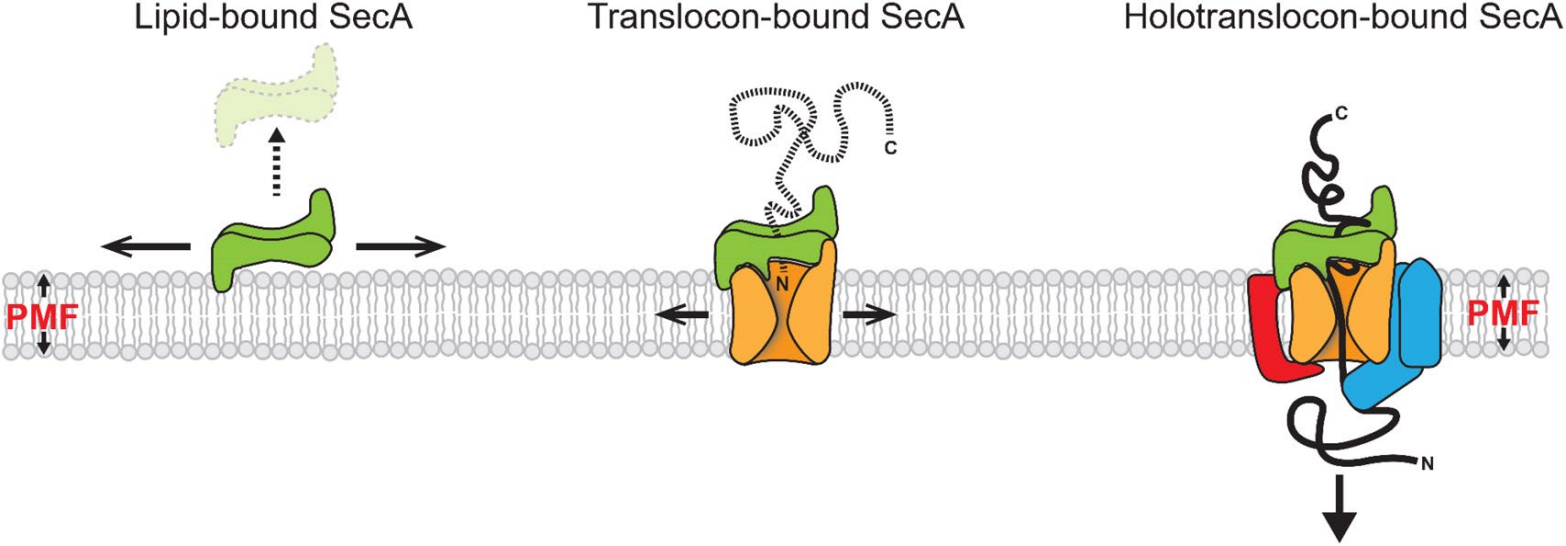
Explanatory model of SecA diffusive populations in living cells. SecA (Green), SecYEG (orange), YidC (red), and SecDF (blue). Based on the diffusion characteristics of SecA observed in *E. coli* cells, and available literature data, we propose that SecA exists in at least three distinct states. (1) A lipid-bound state: SecA is associated at the cytoplasmic membrane in a “scanning” mode, diffusing rapidly along the membrane surface, possibly waiting for substrates to translocate. Although the observations demonstrate that SecA is predominantly associated with the cytoplasmic membrane, a very small cytosolic pool of SecA is also present. (2) A translocon-bound state: SecA exhibit a diffusion rate of a membrane protein. Since SecA does not poses the characteristics of an integral membrane protein, we propose that in this either is an idle or protein translocation engaged state of SecA. SecA is only transiently engaged in this state. (3) A holotranslocon-bound state: the transiently immobile population of SecA is likely bound to a large protein complex, the holotranslocon.

The diffusion coefficients obtained from the particle tracking revealed a highly dynamic nature of SecA under native conditions in cells. By using the cumulative probability distribution of step sizes we found three distinct but interconvertible diffusion rate populations, schematically summarized in Fig. 7. The majority of the SecA diffused with rates corresponding to those of integral membrane proteins (Table 2), representing a subset of SecA molecules possibly bound to SecYEG or the holotranslocon (Fig. 7, translocon-bound SecA). The second population of SecA displayed diffusion rates corresponding to that of cytosolic proteins. This population of molecules is a subset loosely bound to the cytoplasmic membrane and likely “scans” the membrane to bind to SecYEG once loaded with a substrate protein (Fig. 7, Lipid-bound SecA). This observation is in line with biochemical studies showing the interaction of SecA with lipids, in particular anionic phospholipids^26,52^. It was established that SecA is surface-active and penetrates into lipid monolayers containing acidic phospholipids with its N-terminal amphipathic helix^53^, which primes SecA for high-affinity binding to SecYEG^26^. High salt concentrations did not inhibit the insertion, suggesting that the membrane association feature of SecA is not only electrostatic but also a hydrophobic interaction.

The last population showed to be temporarily immobile. These SecA molecules are bound to a very large structure, likely representing the active (holo-)translocon as this population disappeared when translocation is blocked with uncoupler (Fig. 7, holotranslocon-bound SecA). Remarkably, under those conditions, SecA repeatedly but transiently localized to the same spots at the cytosolic membrane, possibly attempting to complete translocation that is interrupted by the loss of the proton-motive force. However, the retention time at those sites is too short to yield a significant immobile population.

In summary, by employing the power of super-resolution microscopy on *E. coli* cells, we were able to gain new insights into the cellular distribution, concentration, oligomeric state and diffusional behavior of the essential SecA ATPase in vivo. Our current findings provide a deeper insight into the dynamics of SecA in living cells and will help to obtain a more detailed molecular understanding of the protein translocation process.

## Materials and methods

### Compounds, bacterial strain and cultivation

Phire Green Hot Start II DNA polymerase and other enzymes were obtained from Thermo Scientific (Waltham, MA). Plasmid DNA and amplicons were purified from gel using the Sigma-Aldrich GenElute Gel Extraction Kit (Sigma-Aldrich, St. Louis, MI) or Zymoclean Gel DNA Recovery kit (Zymo Research, Irvine, CA). Primers were purchased by Sigma-Aldrich and chemicals were obtained from Boom Chemicals (Meppel, Netherlands) and Sigma-Aldrich.

#### Bacterial cultivation

Supplementary Table S1 lists the strains used in this study. *Escherichia coli* K-12 MG1655 was used as a host for the homologous recombination^54^ and subsequent microscopic analysis. *E. coli* strains were grown at 30 °C or 37 °C in Lysogeny-Bertani (LB)^55^, SOB^56^or MOPS EZ rich defined^57^ medium (EZ medium) in shake cultures with appropriate selective markers where needed. When required, transformants were selected on LB agar medium supplemented with 30 µg/ml chloramphenicol, 50 µg/ml kanamycin or 100 µg/ml ampicillin. For λ-red recombinase induction, 27 mM, 40 mM or 60 mM arabinose was used. Growth rates were determined for the *E. coli* strains at 37 °C in MOPS EZ glucose without additional supplements. Optical density at 600 nm was measured every 20 min using a Novaspec Plus spectrophotometer (Amersham, UK). Data was plotted semi-logarithmic and doubling times were calculated using conventional methods.

For fluorescence microscopy, the *E. coli* strains were synchronized by serial dilution. In short, cultures were incubated overnight in LB medium supplemented with appropriate antibiotics. The following day, overnight cultures were diluted 1000-fold in EZ medium supplemented with glucose and appropriate antibiotics for a second overnight incubation. Next, the overnight culture was 300-fold into fresh EZ medium and grown until OD_600_ of ~ 0.4. From this point the cultures were synchronized and were kept growing mid-exponentially by diluting them fourfold with EZ medium every 60 min. Samples for microscopy were withdrawn from these synchronized cultures and imaged at OD_600_ ~ 0.3 to ~ 0.6. To disrupt the protein translocation process, cultures were incubated for 30 min with the appropriate inhibitor. SecA-mediated protein translocation was blocked using either 500 µM or 3 mM NaN_3_. While blocking protein translocation by dissipation of the PMF was achieved via the addition of 5 or 50 µM CCCP.

### Construction of the genomic SecA fusion with fluorescent proteins

Supplementary Table S1 lists the plasmids and primers used in this study. A template plasmid for the mEos3.2 integration fragment was built by ligation of the genes of mEos3.2 and chloramphenicol into the pUC18 vector. In short, Phusion DNA polymerase was used in combination with primers ABS76 and ABS77 to amplify the mEos3.2 gene (753 bp) from pBAD mEos3.2 TEV His10 plasmid and in combination with ABS78 and ABS79 to amplify the chloramphenicol gene (877 bp) from pBAD18 camR plasmid. The resulting amplicons were purified from a 1% agarose gel, digested with AvaI and BamHI (mEos3.2) or BamHI and HincII (chloramphenicol). The amplicons were then cloned into the corresponding sites of pUC18, resulting in pUC18 mEos3.2 camR.

Integration of the yellow protein of electron transfer (Ypet; λ_ex_ = 517 nm, λ_em_ = 530 nm^58^) or the improved photo convertible monomeric Eos3.2 (mEos3.2; Green: λ_ex_ = 506 nm, λ_em_ = 519 nm. Red: λ_ex_ = 573 nm, λ_em_ = 584 nm^59^) gene downstream of the *secA* gene via homologous recombination as described by Datsenko and Wanner^54^. In short, using Phire DNA polymerase with primers sets ABS45 and ABS46 and ABS70 and ABS71, for Ypet and mEos3.2 respectively, 1905 bp and 1649 bp linear DNA integration fragments were obtained. These fragments consisted of a sequence coding for a 14 amino acids long unstructured protein linker, followed by the genes of *Ypet* or *meos3.2* and kanamycin or chloramphenicol resistance. The 5′ and 3′ ends of these fragments were composed of 50 base pairs homologous to the genomic region of interest e.g. the 5′ was homologous to the last 50 nucleotides of *secA* omitting the stop codon to create a translational linked fusion protein, whereas the 3′ was homologous to the 50 nucleotides downstream of the *secA* gene. The integration fragment was gel purified prior to electroporation into competent Lambda Red containing *E. coli* MG1655 cells. After multiple successive screening rounds using primer sets ABS47 & ABS61 (Ypet) and ABS82 & ABS83 (mEos3.2), positive clones were sent for sequencing for verification of correct genomic integration using primers ABS82 & ABS83.

### Immunodetection of SecA-Ypet fusion protein

For verification of the presence of a SecA-Ypet fusion protein by immunodetection, overnight cultures were sonicated and cell debris was spun down at 4000*g* for 10 min at 4 °C. Approximately 40 µg of the resulting lysate was subjected to 10% (w/v) SDS-PAGE gel electrophoresis for Coomassie analysis and blotted on an Immobilon PDVF membrane (0.45 µM) (Merck Millipore, Bedford, MA) using the conventional wet transfer protocol. Immunodetection was carried out with polyclonal anti-SecA antibodies (anti-rabbit, 1:20,000 in PBST + 0.2% I-block) or monoclonal anti-GFP (anti-mouse, 1:2000 in PBST + 0.2% I-block) and subsequent secondary alkaline phosphatase conjugated anti- mouse or anti-rabbit IgG antibodies (1:30,000 in PBST + 0.2% I-block, Sigma). Blots were developed with the CDP-Star chemiluminescence kit (Thermo Scientific, Waltham, MA) and imaged using an ImageQuant LAS4000 (FujiFilm, Tokyo, Japan). Quantification was carried out with ImageJ v1.53b using conventional methods.

### Microscope experimental set-up

In-vivo microscopy measurements were performed at 37 °C on an Olympus IX-81 microscope equipped with an automated z-drift compensator and a 100 × total internal reflection fluorescence (TIRF) objective (UApoN, NA 1.49 (oil), (Olympus, Center Valley, PA) set to epi-illumination (ϴ < ϴ_c_). Imaging of the molecules was carried out in a mid-cell focal plane, which was autocorrected for z-drift during acquisition. Ypet molecules were excited by a 514 nm continuous wave (CW) laser (Coherent, Santa Clara, CA) at ~ 1.39 kW·cm^−2^. Imaging of mEos3.2 was accomplished by photo converting mEos3.2 from green to red emission by exciting molecules for 5 s with a 405 nm CW laser line at ~ 150 W·cm^−2^, after which molecules were excited by a 561 nm CW laser line at ~ 350 W·cm^−2^. Fluorescent emissions were detected after passage through an emission filter (Ypet; emission filter: 540/30 and mEos3.2; emission filter: 645/75 (Chroma, Bellows Falls, VT)). Frames were captured using MetaVue imaging software (Molecular Devices, Sunnyvale, CA) via an 512 × 512 pixel electron multiplying charge coupled device (EMCCD) camera (C9100-13, Hamamatsu, Hamamatsu City, Japan) with EM-gain set to 1200 × at 33 frames·s^−1^ and 100 frames·s^−1^ for kymograph analysis and single particle tracking.

To optimize the bacterial cell conditions during microscopy, e.g. provide enough oxygen for proper maturation of the fluorescent proteins and maintain exponential growth, all experiments were carried out in homebuilt flow cells. High precision coverslips (75 × 25 × 0.17 mm) were functionalized with 3-aminopropyltriethoxysilane (APTES, Sigma) to enable non-toxic cell adherence^60^. In short, coverslips were sonicated in 5 M KOH for 45 min at 30 °C. After thorough rinsing with double-distilled H_2_O, the slides were dried for 30 min at 110 °C. Next, the coverslips were plasma cleaned (PE-50, Plasma Etch, Inc) for 10 min and directly after, incubated in 2% APTES (v/v) in acetone. These functionalized coverslips were stored under vacuum in a desiccator to slow down the silane degradation. Prior to each microscopy experiment, a channel was fixed and capped by a plasma cleaned object slide containing inlet and outlet tubes. Synchronized cells growing mid-exponentially were flushed through and after a short settling time, oxygen rich EZ-glucose medium was flowed through the flow cell at 30 µL·min^−1^ after which fluorescence acquisition was started. For impaired protein translocation conditions, appropriate concentrations of NaN3 or CCCP was added to the EZ-glucose medium to prevent cells from recovering from the impaired state.

### Data analysis

Data obtained from the microscope measurements were analysed with ImageJ v1.48 using built-in and purpose-built plugins. Movies were corrected for electronic offset and background fluorescence prior to analysis. Cells were selected using ROIs obtained by the built-in “Threshold” function, manually adjusted for best cell selection. To visualize the boundaries of the bacterial cell, fluorescence intensities of the first 7.5 s were averaged and plotted as a single image using the built-in function “Z-project” of ImageJ. For super-resolution reconstructions, cellular distribution, copy number determination, foci intensity and single particle tracking, the purpose-built plugin SURREAL was used.

Cell volumes were calculated using the cellular features e.g. width and length, determined from the ROI, in a conventional rod-shaped bacterium model^61^. Data was visualized using OriginPro v9.1 (OriginLab Corporation, Northampton, MA) or MATLAB R2016b (The MathWorks, Natick, MA).

#### Peak detection

When emission light from a fluorescent protein is recorded by an EMCCD camera, the resulting signal has a certain point-spread function (PSF) which can be defined by a symmetrical Gaussian function given by:

2 $$f\left( {x,y} \right) = A e^{{ - \frac{{\left( {x - x_{0} } \right)^{2} + \left( {y - y_{0} } \right)^{2} }}{{2\sigma^{2} }}}}$$

where A is the amplitude, $x_{0}$,$y_{0}$ the centroid coordinates of the peak and $\sigma$ the symmetrical spread of the signal. To detect SecA beyond the diffraction limit, images were processed using a discoidal averaging filter with an inner and outer radius of respectively 1 and 3 pixels^62^. Subsequently, local maxima were selected on a minimal distance between peaks of 3 pixels and a minimal intensity using a dynamic threshold defined as $\overline{x} + n*\sigma$, where *n* is indicated in the text, peaks not fulfilling these criteria were discarded. Next, a two-dimensional Gaussian model Eq. (2) was fitted to each PSF on the original unprocessed image by minimizing the sum of squares of the residuals by means of the Levenberg–Marquardt algorithm^63,64^. The resulting Gaussian model gave the amplitude, sub-pixel coordinates, symmetrical spread and localization accuracy of the peak positions for each frame.

#### Super-resolution reconstruction the SecA ATPase

To create a super-resolution image of SecA, local maxima were detected using a threshold of *n* = 2. A normalized Gaussian distribution with a standard deviation equal to the localization error for each fitted peak was plotted in a color-coded image to obtain a super-resolution reconstruction. Here the red colors indicate a low localization accuracy and/or frequency and white indicates a high localization accuracy and/or frequency.

#### Cellular distribution of the SecA ATPase

The specific localization of a membrane or cytosolic protein results in a typical short-axis cross section profile, which can be used as compartmental markers to deconvolve a profile of interest following Eq. (3). Rewriting this formula to Eq. (4), we obtain a function of which the outcome is the cytosolic weighing factor by which we can determine the profile of interest’s cellular distribution. To determine the cellular distribution of SecA, fluorescence signals from an image sequence spanning the first 7.5 s were averaged and a short-axis cross section was created for bacteria expressing either the lactose transporter LacY fused to eYFP as an inner membrane protein (IMP), Ypet as cytosolic marker (CP) or SecA-Ypet. Cross-section data of 20 cells per data set were averaged and normalized to a maximum of 1. The resulting averaged cross-section profiles were used in Eq. (4) to deconvolve the SecA profile to obtain the distribution ratios of SecA, e.g. α, cytosolic, and β, membrane bound fractions

3 $$profile_{SecA} = \alpha \cdot profile_{CP} - \beta \cdot profile_{IMP}$$

4 $$\alpha = \frac{{ - profile_{CP} + profile_{SecA} }}{{ - profile_{CP} + profile_{IMP} }}$$

#### Determining the average single-molecule intensity

For determining single fluorophore intensities, a purpose-built plugin was used. Measuring single fluorophore intensities started when fluorescent molecules were spatially well separated, e.g. frames 100–1000, to minimize errors in determining the intensity. Local maxima were detected using the ImageJ built-in “Find maxima” function with a fixed minimal noise tolerance based on the intensities observed in the last frames. Signals passing the tolerance threshold were selected with a radius of 2 pixels from the centroid. Foci fulfilling the criteria were fitted with a 2D Gaussian using Eq. (2), best fitting parameters were obtained by minimizing the sum of squares of the residuals by means of the Levenberg–Marquardt algorithm. Subsequent fitting data was filtered on a minimal adjusted R^2^ above 0.75, to obtain fluorescence intensity data from in focus fluorophores. Next, amplitudes and point-spread functions (PSF) of the filtered data were used in Eq. (5) to calculate the single-molecule integrated Gaussian intensity. Average single-molecule intensities were obtained by plotting the calculated integrated Gaussian intensities in distributions with a bin size (W) obtained from Eq. (6)^65^. Fitting a Gaussian function to the distribution resulted in a centroid value representing the raw integrated gray value of a single Ypet or mEos3.2 molecule.

5 $$\mathop \smallint \limits_{ - \infty }^{\infty } \mathop \smallint \limits_{ - \infty }^{\infty } f\left( {x,y} \right)dxdy = 2 \pi A\sigma_{x} \sigma_{y}$$

6 $$W = 3.49\sigma n^{{ - \frac{1}{3}}} .$$

#### Cellular concentration of the SecA ATPase

Excitation of the fluorophores by epi-illumination (ϴ < ϴ_c_) with a high-power density results in fluorescence emission from all the mature Ypet or mEos3.2 molecules. Integrating the total fluorescence emission within a cell, represent the total quantity of SecA molecules per cell. Dividing this number by the integrated gray value of a single Ypet or mEos3.2 molecule, resulted in the SecA copy number per cell or when divided by the cell volume in SecA copy number per femtoliter (µm^3^).

#### In vivo oligomeric state of the SecA ATPase

For determining the oligomeric state of SecA in living cells, foci were detected in each frame using a similar approach as described in the peak detection section. Only here, instead of a dynamic threshold a minimal fixed gray value was chosen for peak detection based on the fluorescence intensities of molecules in the last frames. The obtained sub-pixel coordinates after peak fitting were used to create a selection with a radius of 2 pixels from the centroid around the PSF in the unprocessed image, from which the raw integrated density was calculated and divided by the integrated Gaussian intensity of a single-molecule to obtain the number of molecules per focus.

#### In vivo membrane diffusion rates of the SecA ATPase

For kymograph analysis, frames were filtered with a discoidal filter to increase the SNR. Next, a manual selection of the membrane was made based on an average fluorescence z-projection. This selection was applied to the SNR enhanced movie, after which the membrane on each frame was straightened using the built-in function “Straighten” of ImageJ. The resulting straightened cell membrane consisted of a certain length and width in pixels. For each frame and pixel along the length of membrane, the intensities of the corresponding pixels in width were averaged and subsequently plotted in the kymograph. Since not all cells are of the same length, polar coordinates between 0 and 2π were used to simplify the location in the membrane.

To study the diffusive behaviour of SecA, particles were detected using a dynamic threshold value of *n* = 5. The fitting data was filtered on a minimal adjusted R^2^ exceeding 0.2, where after the coordinates were used in particle tracking by linking two particles located nearest to each other in consecutive frames. A maximum step size constraint of 6 pixels was used to prevent linkage of particles too far apart to be the same. The resulting step size based trajectory data was used in MSD analysis and as input for a purpose-built MATLAB script for calculation of the cumulative probability distribution (CPD) of step sizes. To obtain a MSD based diffusion coefficient with the highest accuracy possible, only the first four data points were fitted using the Brownian motion model Eq. (7) to describe the observed movement.

7 $$r^{2} = 2nD\tau$$

where n is the number of dimensions and D the diffusion coefficient for each time point (τ). Heterogeneity in the diffusion behaviour of the particles tracked, was analysed by the CPD of the step sizes. The CPD is defined as the probability of a molecule staying within an area defined by a radius, r, after a given time, τ. Step size data obtained from high temporal resolution measurements of single cells was aggregated, of which a probability density function (PDF) was created based on a 1 nm bin size. Normalizing the PDF results in the CPD, to which the cumulative probability distribution function (CPF) was fitted. Homogeneous diffusion particles can be described by a single exponential CPF (Eq. 8). The diffusion coefficient is defined as D and is influenced by the localization accuracy σ.

8 $$CPF\left( {r^{2} ,\tau } \right) = 1 - e^{{\left( {\frac{{ - r^{2} }}{{4D\tau + 4\sigma^{2} }}} \right)}}$$

The experimental localization accuracy σ was determined for the mean X and Y localization errors, parameters resulting from the Levenberg–Marquardt LSF algorithm. Experimental obtained step size data with high temporal resolution was fitted to a single, double, triple and quadruple exponential CPF model. The goodness-of-fit was determined for each model by calculating the residual sum of squares (RSS). The model which fitted best, e.g. RSS closed to 0, was used to calculate the diffusion coefficient for each population from the slope using Eq. (7) by plotting the obtained MSD values as a function of time.

## Supplementary Information

Supplementary Information
