# Supplementary material for: Cellular dynamics of the SecA ATPase at the single molecule level
Source: Sci Rep. 2021 Jan 14;11:1433. doi: 10.1038/s41598-021-81081-2 (PMC7809386; doi:10.1038/s41598-021-81081-2)
Supplement: Supplementary file 1 — Supplementary Information [file 41598_2021_81081_MOESM1_ESM.docx]

**Cellular dynamics of the SecA ATPase at the single molecule level**

Anne-Bart Seinen^1,2^, Dian Spakman^1^, Antoine M. van Oijen^3^, Arnold J. M. Driessen^1*^

^1^ Department of Molecular Microbiology, Groningen Biomolecular Sciences and Biotechnology Institute, and the Zernike Institute for Advanced Materials, University of Groningen, Groningen, The Netherlands

^2^ Current address: AMOLF, Science Park 104, 1098 XG, Amsterdam, Netherlands

^3^ School of Chemistry, University of Wollongong, Wollongong, Australia

**Supplementary tables and figures**

Supplementary Table 1: Strains, plasmids and primers

|  | **Genotype/relevant features** | **References** |
| --- | --- | --- |
| **Strains** |  |  |
| *E. coli* MG1655 | K-12, F^–^ λ^–^ *ilvG*^–^ *rfb-50 rph-1* | Lab collection |
| *E. coli* MG1655 SecA-Ypet | MG1655, *secA*::*ypet* | This work |
| *E. coli* MG1655 SecA-mEos3.2 | MG1655, *secA*::*meos3.2* | This work |
| **Plasmids** |  |  |
| pKD46 | Lambda Red expression vector | Wanner et. al. ^48^ |
| 11AAWJ2P Ypet knock in cassette | Template for linear Ypet integration DNA fragment | van Oijen et. al. |
| pBAD mEos3.2 TEV His10 | Template for mEos3.2 gene | Van Oijen et. al. |
| pUC18 mEos3.2 camR | Template for linear mEos3.2 integration DNA fragment | This work |
| pBAD18 CamR | Template for chloramphenicol gene | Lab collection |
| pUC18 | Backbone for insertion of mEos3.2 and chloramphenicol gene | Lab collection |
| **Primers** | **Sequence 5`-3`** | |
| ABS45 SecA_C-ter_Ypet_For | GCGGTTCTGGTAAAAAATACAAGCAGTGCCATGGCCGCCTGCAACGCCTGCAAAGCAGCGCTGGCAGCGCGGCGGGCAGCGCGAATATGTCTAAAGGTGAAGAATTATTCACTGGTG | |
| ABS46 SecA_C-ter_Ypet_Rev | CTATAAAAAAGGCGCAGAATCCTGCGCCTTTTACTTCAACAGTTAGCTTGAAGTTCCTATTCTCTAGAAAGTATAGGAACTTCG | |
| ABS47 SecA-Ypet_Seq_For | GCGCAAATGCAGCAGCTTAG | |
| ABS61 ColPCR_KanR_Rev | CTTGCCATCCTATGGAACTG | |
| ABS70 SecA_C-Ter_mEos_For | GCGGTTCTGGTAAAAAATACAAGCAGTGCCATGGCCGCCTGCAACGCCTGCAAAGCAGCGCTGGCAGCGCGGCGGGCAGCGCGAAT | |
| ABS71 SecA_C-Ter_mEos_Rev | CTATAAAAAAGGCGCAGAATCCTGCGCCTTTTACTTCAACAGTTAGCTTGAAGTTCCTATTCTCTAGAAAGTATAGGAACTTCG | |
| ABS76_mEos3.2_Link_AvaI_For | GTAGTAGTACCCGGGCGCCTGCAAAGCAGCGCTGGCAGCGCGGCGGGCAGCGCGAATATGAGTGCGATTAAGCCAGAC | |
| ABS77_mEos3.2_Link_BamHI_Rev | GTAGTAGTAGGATCCTTATCGTCTGGCATTGTCAGG | |
| ABS78_CamR_FRT_BamHI_For | GTAGTAGTAGGATCCGAAGTTCCTATTCTCTAGAAAGTATAGGAACTTCCGCCCCGCCCTGCCACTCATC | |
| ABS79_CamR_FRT_HincII_Rev | GTAGTAGTAGTCGACGAAGTTCCTATTCTCTAGAAAGTATAGGAACTTCGATCGGCACGTAAGAGGTTC | |
| ABS82 SecA Seq For | CGTTTAGCGCAAATGCAGCAGCTTAG | |
| ABS83 SecA Seq Rev | CAGTTTATTCGCCATGTGCGCATCTG | |


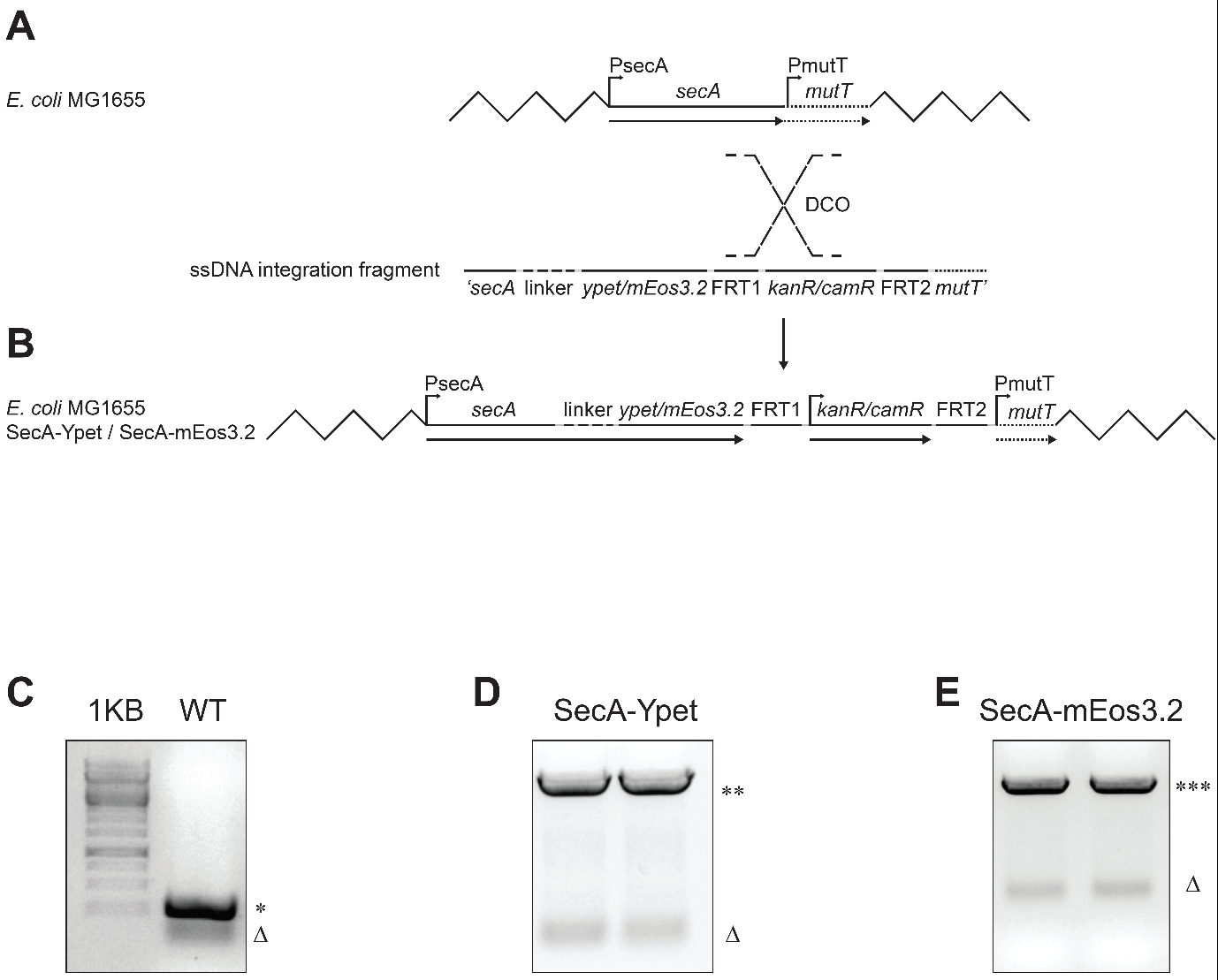


Supplementary Fig. 1 - *secA* gene fusion strain construction and chromosomal integration. Homologous recombination of a ssDNA integration fragment into the *secA* locus of *E. coli* K12 MG1655 strain, creating the SecA-Ypet or SecA-mEos3.2 fusion strains. (**A**) Genomic map of the locus in *E. coli* K12 MG1655 strain. (**B**) Genomic situation of the *secA* locus after successful homologous recombination of the single stranded DNA integration fragment, leading to a fusion of the Ypet or mEos3.2 gene in frame with the *secA* gene still under its native promotor. The correct integration into the *secA* locus yielding the SecA-FP fusion proteins was confirmed by colony PCR (**C-E**). (**C**) Colony PCR using ABS82 and ABS83 primers of the wild-type situation results in a 317 bp fragment (*). (**D**) Correct integration of the Ypet knock-in cassette results in a 2114 bp fragment (**). (**E**) the mEos3.2 cassette yields an 1870 bp fragment (***). Primers are indicated by delta (Δ).


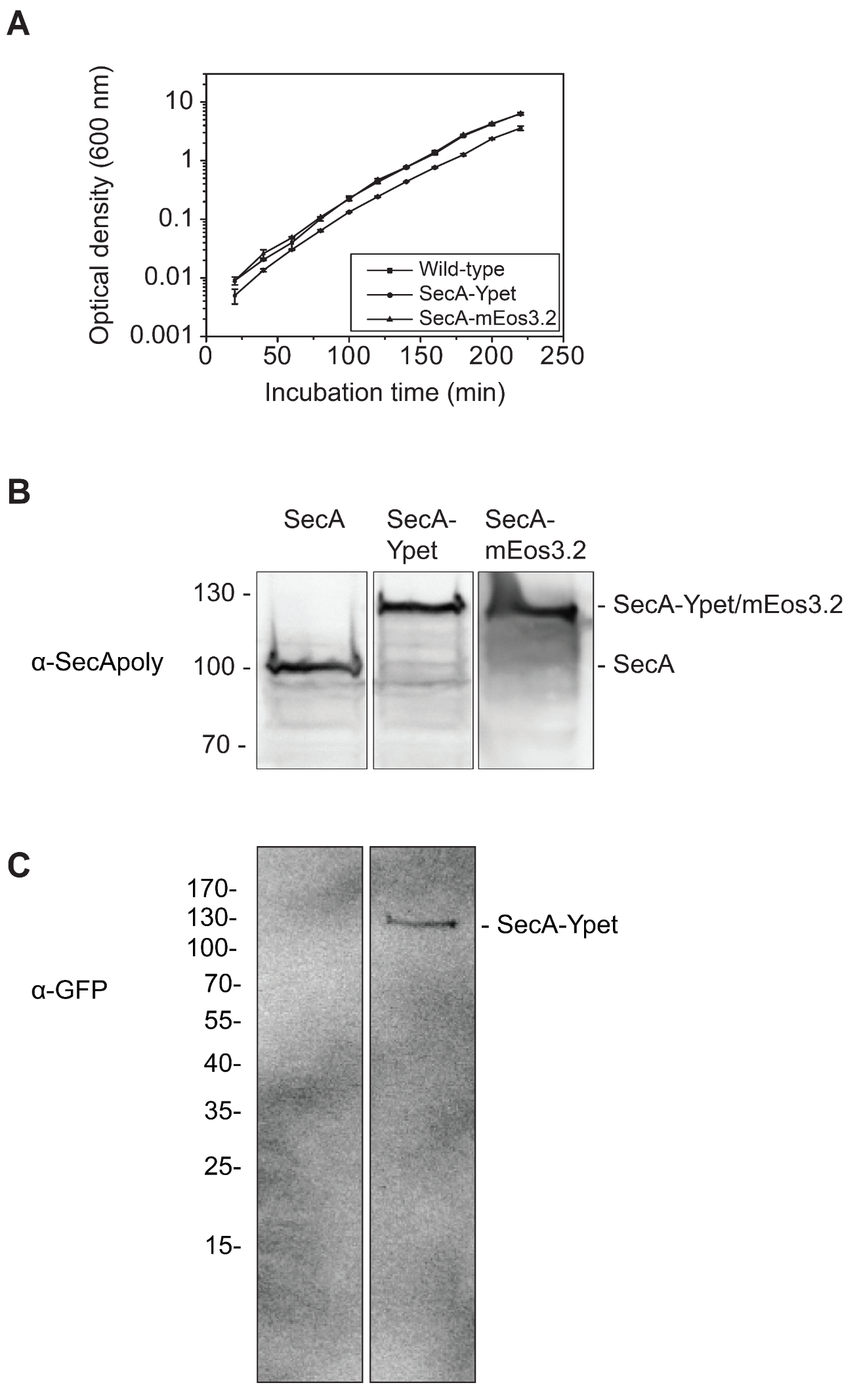


Supplementary Fig. 2 - C-terminal SecA-FP fusions do not interfere with the SecA activity and successfully replaced the endogenous SecA. (**A**) Growth rates of E. coli MG1655 (■) versus the SecA-Ypet (●) and SecA-mEos3.2 (▲) fusion strains plotted against a logarithmic axis. The growth curves display a similar shape, indicating a comparable growth between the E. coli MG1655 wild-type strain and the constructed SecA mutant strains. (**B**) The SecA fusion proteins replaced the endogenous SecA. Western blot of SecA wild-type, SecA-Ypet and SecA-mEos3.2 synthesis in E. coli MG1655 strains. Indicated with SecA, lysate from E. coli MG1655 expressing wild-type SecA. A band is visible at the expected size of approx. 100 kDa. Indicated by SecA-Ypet and SecA-mEos3.2 are the lysates obtained from the E. coli MG1655 strains expressing SecA-Ypet and SecA-mEos3.2. Bands at the expected size of approx. 130 kDa are clearly visible indicating the presence of the SecA fusion constructs. No wild-type SecA bands are observed in these lanes, the fragments visible in all lanes are due to unspecific binding of the polyclonal SecA antibody. Identical band intensities suggest similar expression levels between the strains. Supplemental Figure 3 shows the uncropped western blots. (**C**) Western blot of SecA-Ypet lysate with GFP antibody SecA-Ypet lysate immunoblotted with antibodies raised against GFP. Only one band corresponding to the SecA-Ypet fusion protein is visible at approximately 130 kDa. The absence of other bands indicates that the fusion protein is not cleaved.


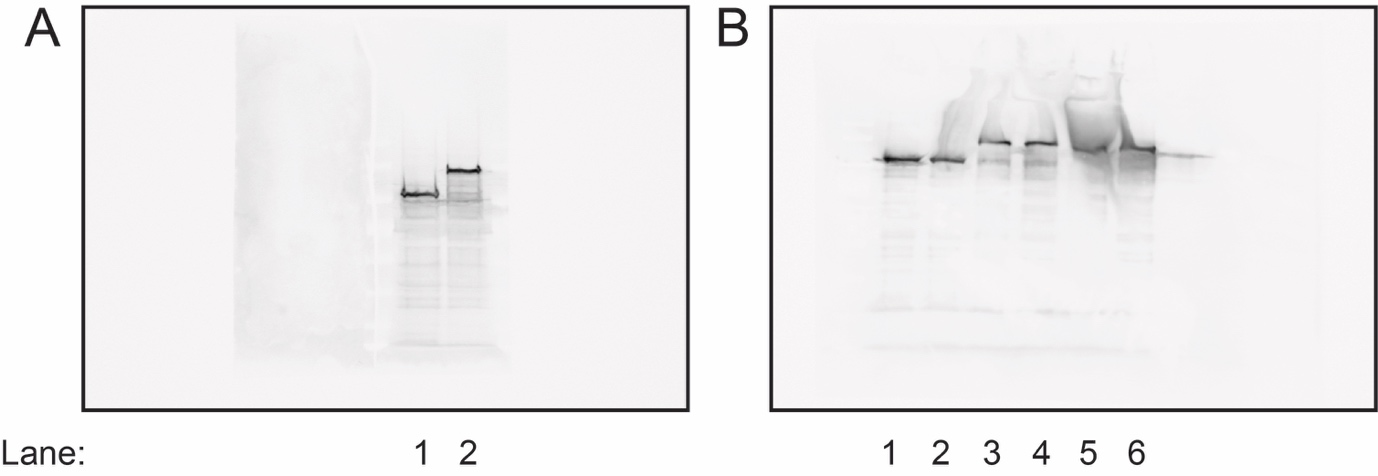


Supplementary Fig. 3. Uncropped Western blots of lysates from *E. coli* MG1655 expressing SecA wild-type, Sec-Ypet and SecA-mEOS3.2 developed with a polyclonal SecA antibody. (**A**) Western blot linked to Supplemental Figure 2B showing the wild type SecA and SecA-Ypet MG1655 cells. Lane 1: Wild-type SecA; Lane 2: SecA-Ypet. (**B**) Western blot linked to Supplemental Figure 2B showing the SecA-mEOS3.2 MG1655 cells. Lane 1 and 2: Wild-type SecA; Lane 3 and 4: SecA-Ypet; Lane 5 and 6: SecA-mEOS3.2.


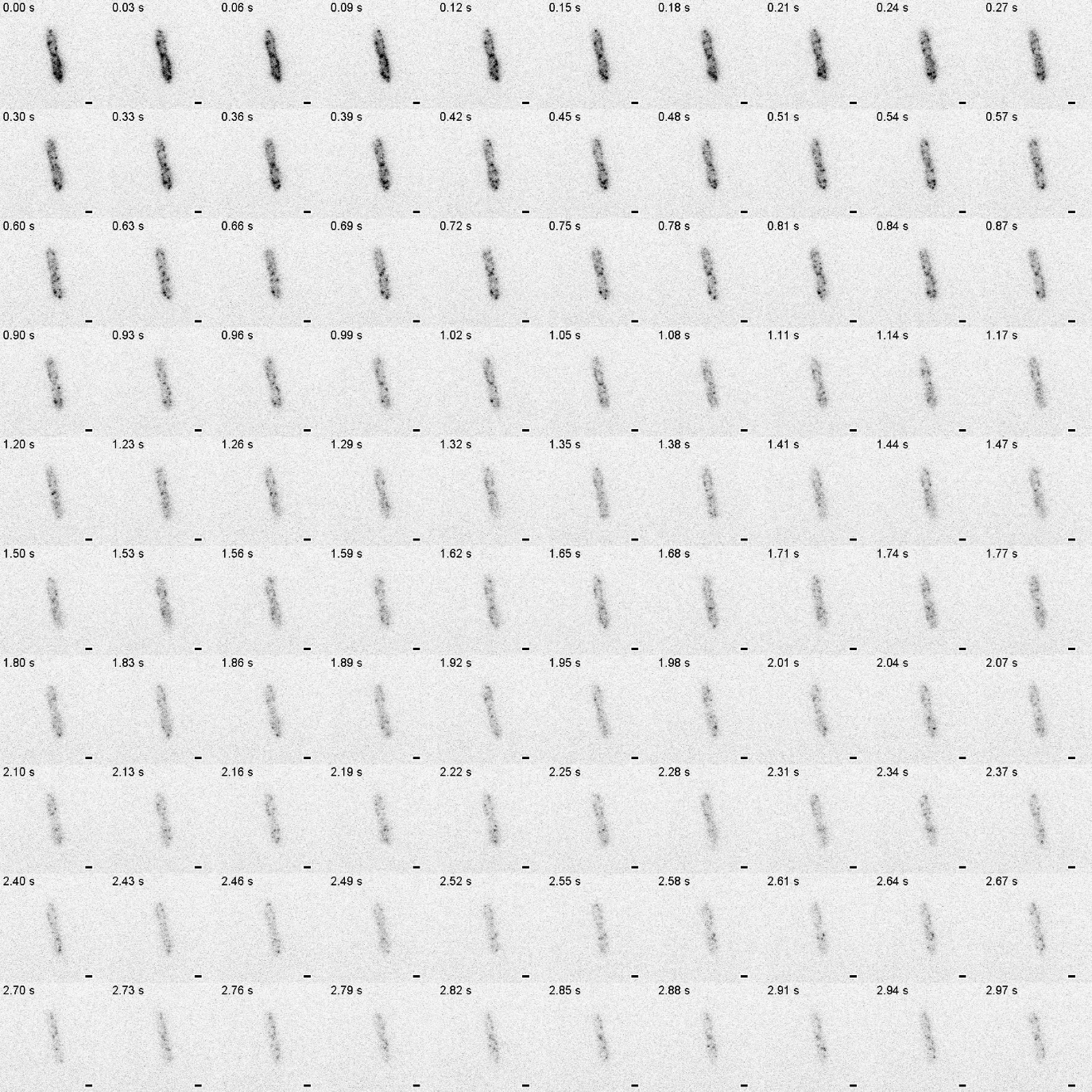


Supplementary Fig. 4 – *In-vivo* visualization of SecA-Ypet. Unprocessed microscopy data of the first 3 seconds or 100 frames during excitation. Scale bar is 1 µm.


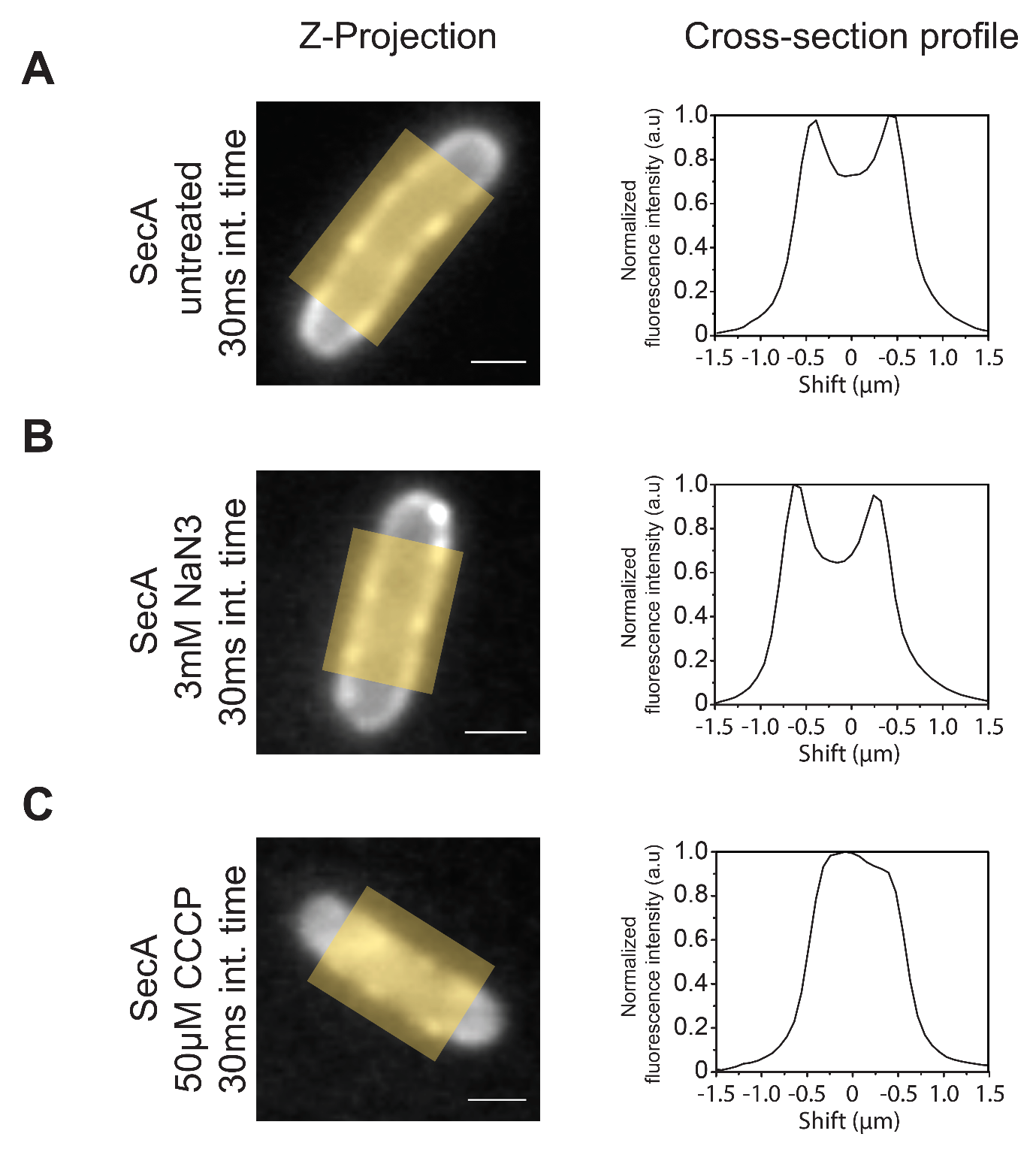


Supplementary Fig. 5 – Cross-section profile selections. Indicated by the yellow bar in (**A**),(**B**)and(**C**) are the example selections to obtain cross-section profiles of the cells. The pixel values across the long-axis were averaged to obtain a general short-axis cross-section profile. Scale bar is 1 µm.


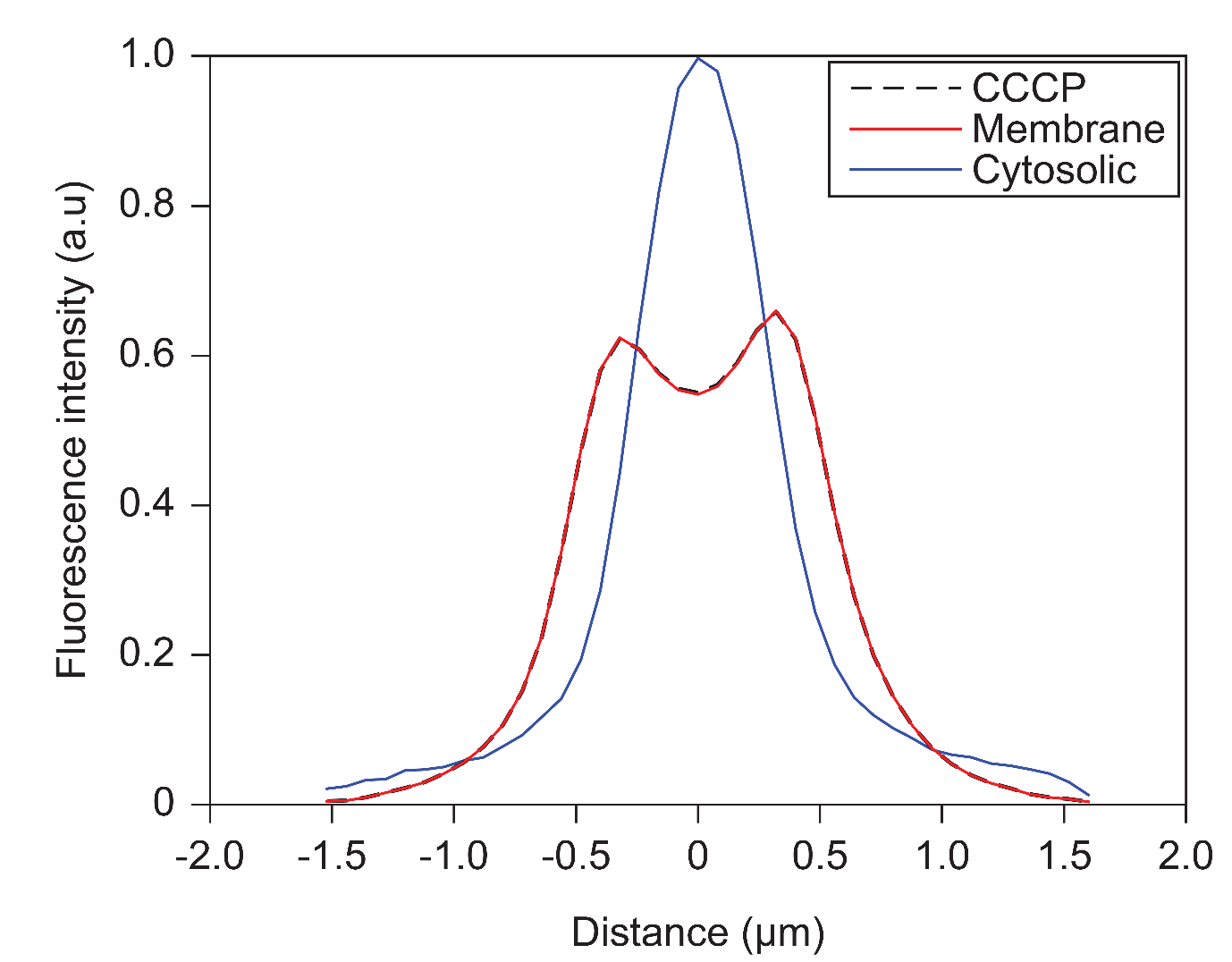


Supplementary Fig. 6 – Cellular distribution profiles of the fluorescence for the membrane protein LacY-Ypet (Membrane, red solid line), cytosolic protein Ypet (Cytosolic, blue solid line) and LacY-Ypet treated with 50 µM CCCP (Black dashed line).


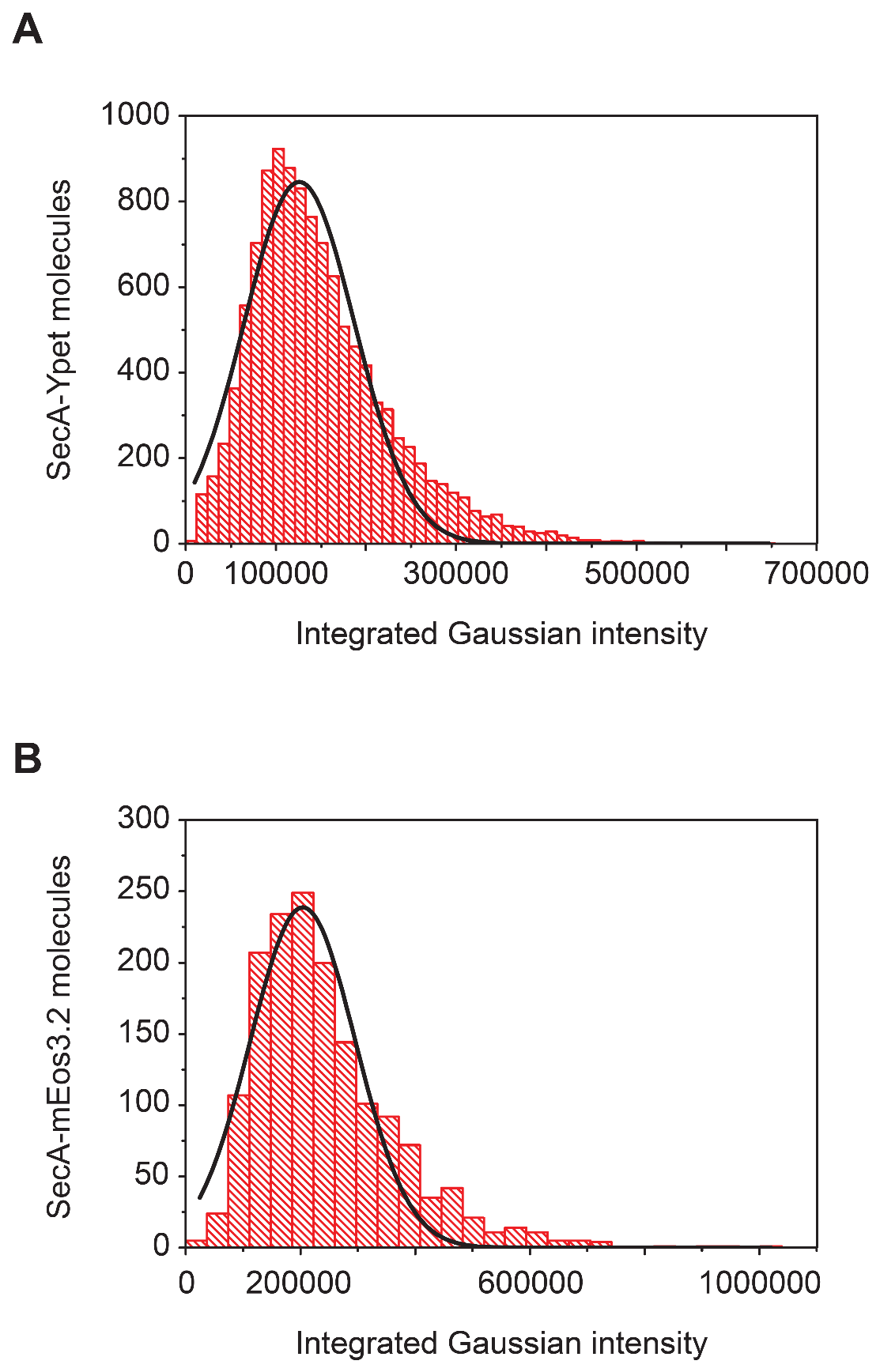


Supplementary Fig. 7: Integrated Gaussian intensity distributions of SecA-Ypet (**A**) and SecA-mEos3.2 (**B**). Integrated Gaussian intensities were plotted with bin size obtained from Eq.5. 2D Gaussian fitting resulted in centroid integrated density value of ~125895 (adjusted R^2^ 0.947) for SecA-Ypet and ~204020 (adjusted R^2^ 0.933) for SecA-mEos3.2, corresponding to the fluorescence intensity of a single molecule. The single molecule intensity values obtained were recalculated for each new experiment. Identical peak values were obtained from multiple experiments.


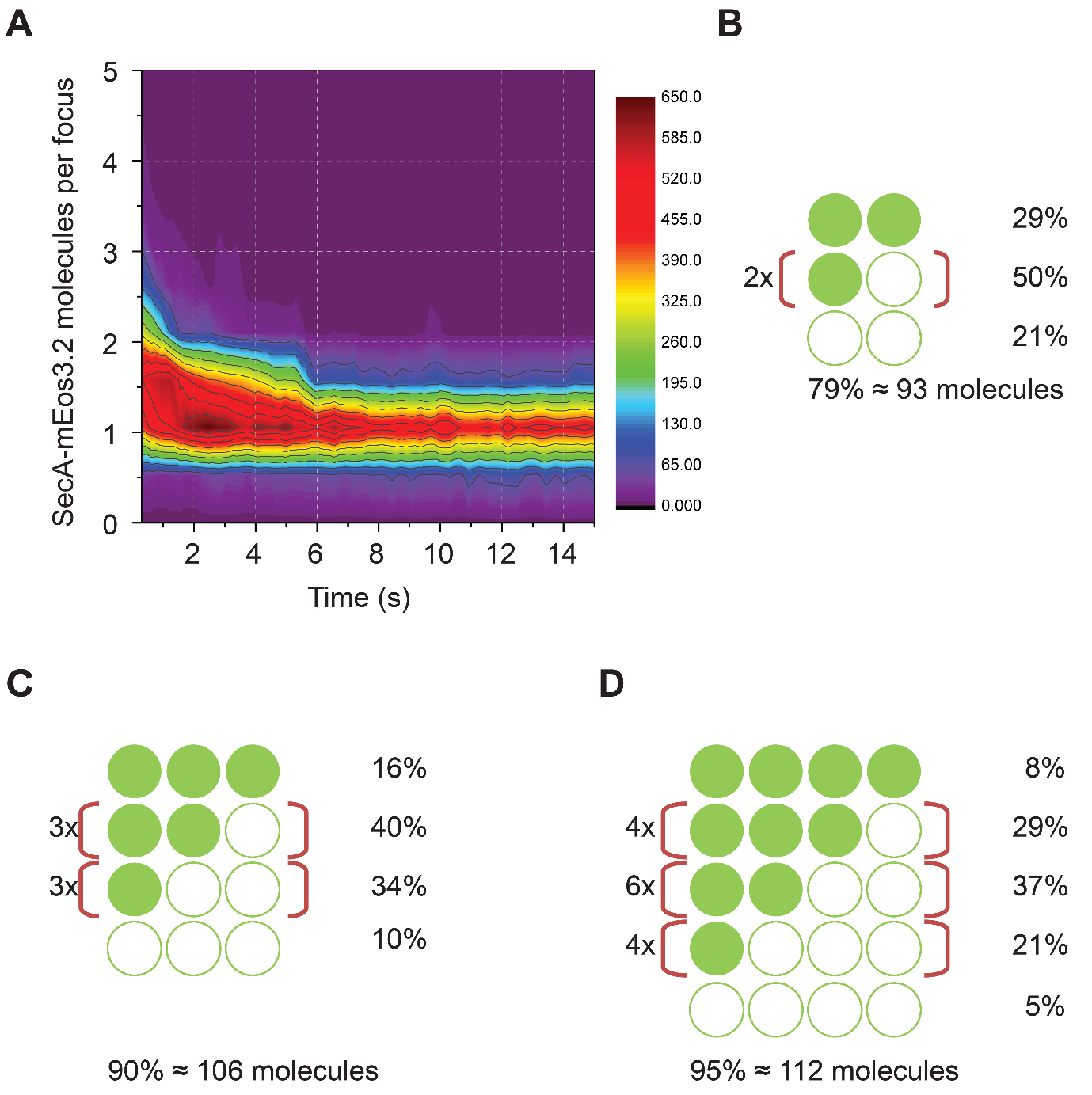


Supplementary Fig. 8 - Dimerization of SecA is confirmed by the SecA-mEos3.2 fusion construct. (**A**) SecA-mEos3.2 heat map displaying the number of SecA molecules per focus. Due to the switching efficiency dimeric foci are observed rarely. (**B-D**) k-combinations giving the maximum number of molecules observable for di-, tri and tetrameric SecA states assuming 54% switching efficiency and a copy number of 118 molecules. (**B**) Dimeric SecA-mEos3.2 situation, given the photo conversion efficiency, 79% or 93 molecules are visible in optimal conditions and 21% or 25 molecules are undetectable. This number is lies in the range of the observed copy number of SecA-mEos3.2 (64$\pm14$), indicating a dimeric state of SecA. Additional evidence comes from assuming tri- or tetrameric states, where respectively 90% and 95% of the molecules would be detectable. Since these numbers are in the range of the detected copy number with Ypet, it is highly unlikely that SecA in the native form forms higher oligomeric states than the dimer under native conditions.


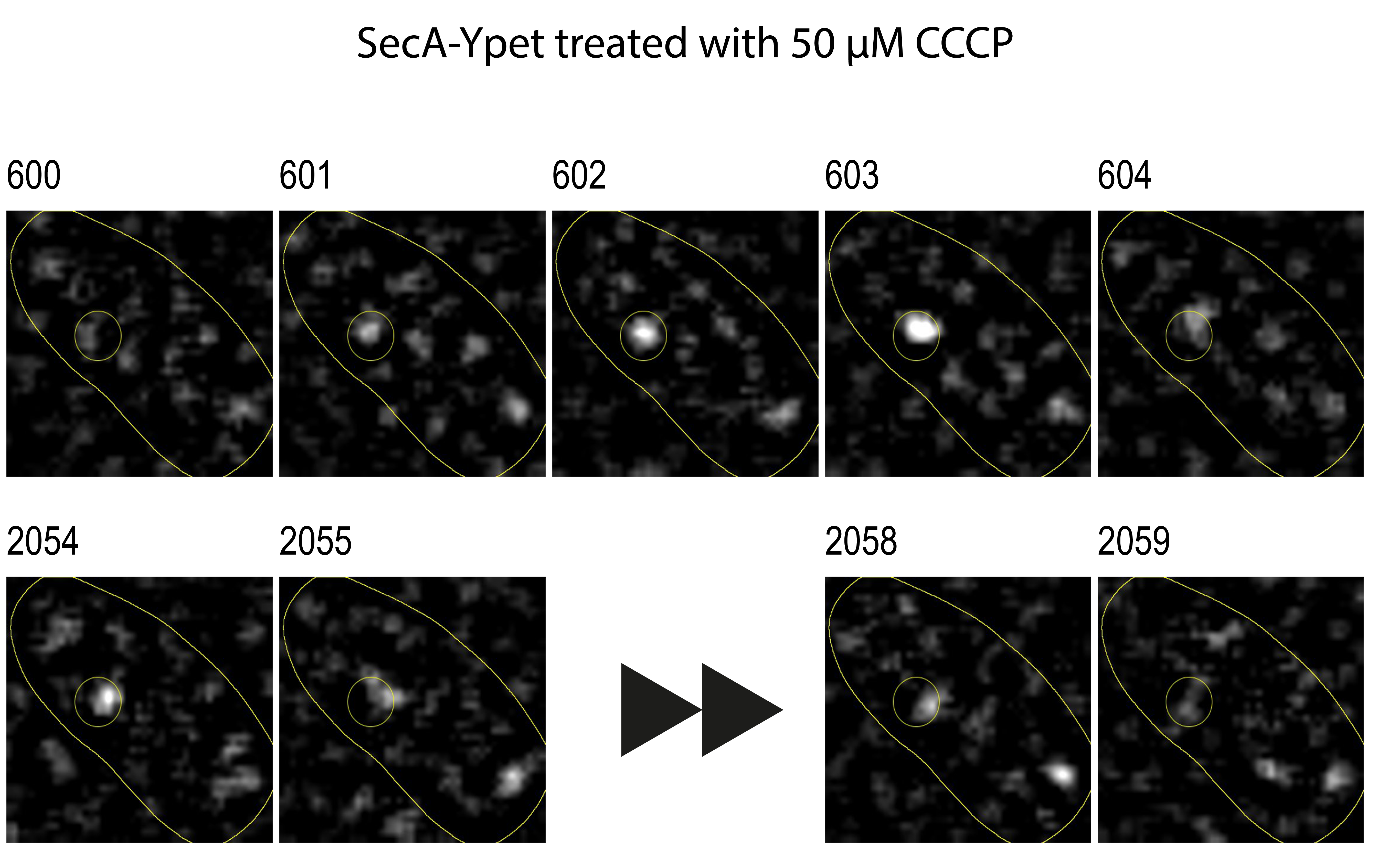


Supplementary Fig. 9 - Reoccurrences of SecA-Ypet to the same location. Image sequence of SecA-Ypet with a rough indication of the cell outline (yellow outline) and location of reoccurrences (yellow circle). Image is processed with a discoidal averaging filter to enhance SNR for visualization.
